# Noncovalent Enzyme Nanogels via a Photocleavable Linkage

**DOI:** 10.1021/acs.macromol.2c01334

**Published:** 2022-11-03

**Authors:** Neil L. Forsythe, Mikayla F. Tan, Daniele Vinciguerra, Jacquelin Woodford, Adam Z. Stieg, Heather D. Maynard

**Affiliations:** † Department of Chemistry and Biochemistry, University of California, 607 Charles E. Young Drive East, Los Angeles, California 90095, United States; ‡ California NanoSystems Institute, 570 Westwood Plaza Building 114, Los Angeles, California 90095, United States

## Abstract

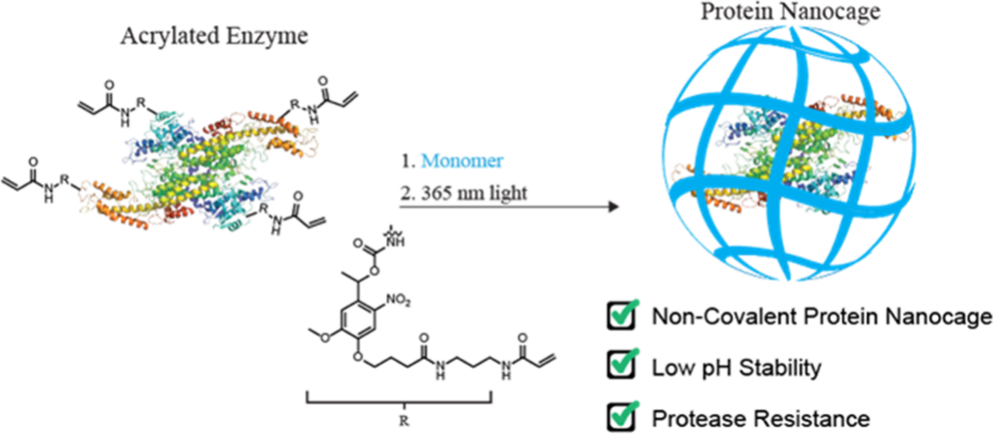

Enzyme nanogels (ENGs) offer a convenient method to protect therapeutic proteins from in vivo stressors. Current methodologies to prepare ENGs rely on either covalent modification of surface residues or the noncovalent assembly of monomers at the protein surface. In this study, we report a new method for the preparation of noncovalent ENGs that utilizes a heterobifunctional, photocleavable monomer as a hybrid approach. Initial covalent modification with this monomer established a polymerizable handle at the protein surface, followed by radical polymerization with poly(ethylene glycol) methacrylate monomer and ethylene glycol dimethacrylate crosslinker in solution. Final photoirradiation cleaved the linkage between the polymer and protein to afford the noncovalent ENGs. The enzyme phenylalanine ammonia lyase (PAL) was utilized as a model protein yielding well-defined nanogels 80 nm in size by dynamic light scattering (DLS) and 76 nm by atomic force microscopy. The stability of PAL after exposure to trypsin or low pH was assessed and was found to be more stable in the noncovalent nanogel compared to PAL alone. This approach may be useful for the stabilization of active enzymes.

## Introduction

Enzymes are utilized extensively in a scope of industries including the production of detergents, sensors, sweeteners, textiles, cosmetics, and pharmaceuticals.^1^ For biocatalysis applications, the inherent specificity of enzymes along with their high turnover rates makes them attractive tools for the synthesis and production of structurally complex molecules. Furthermore, enzymes have also become increasingly important as therapeutics. For instance, enzyme replacement therapies (ERTs) are successful in treating a number of lysosomal storage diseases and immunodeficiencies.^2^ While these enzymatic approaches to solving problems in both organic chemistry and medicine have many merits, the use of enzymes as such is not without their challenges.

Enzymes, like many proteins, are often unstable. External factors such as temperature, organic solvents, pH, mechanical agitation, proteases, and light can all lead to irreversible denaturation or inactivation of enzymes.^3^ In both biocatalysis and ERT applications, these limitations are often prohibitive. Biocatalytic processes often require organic co-solvents, agitation, or temperature variation to support sufficient product formation, while in vivo application of therapeutic enzymes can suffer from immune-mediated clearance, protease degradation, and/or renal clearance. To combat this, a number of strategies have been developed to help increase enzyme stability.^4^ For instance, covalent attachment of functional polymers such as poly(ethylene glycol) (PEG), poly(N-(2-hydroxypropyl)methacrylamide), polysaccharide derivatives, and poly(N-isopropylacrylamide) have proved effective at increasing the stability of enzymes to stressors including heat, pH change, and protease degradation.^5,6^ Beyond the direct conjugation of linear polymers, a variety of methods to increase enzyme stability have been pursued, such as site-directed mutagenesis to remove degradation-prone regions from the protein structure,^7^ covalent or noncovalent immobilization onto a matrix,^8,9^ and direct encapsulation within nanoparticles, liposomes, or micelles.^4^ Typically, immobilization and encapsulation favor larger complexes composed of multiple enzymes, which can constrain substrate diffusion and/or lead to protein aggregation. An approach that favors smaller sizes and individual encapsulation is enzyme nanogels (ENGs) that can offer greater colloidal stability for encapsulated proteins and controlled substrate diffusion.^10^

Fundamentally, the synthesis of ENGs involves the localization of monomers and crosslinkers around the surface of the protein followed by polymerization to yield a protective, polymeric shell.^11^ First-generation nanogels involved covalent attachment of acryloyl groups to the lysine residues of the protein, followed by polymerization of acrylamide monomers.^12,13^ This strategy has been effectively expanded to a number of enzymes including chymotrypsin,^14,15^ carbonic anhydrase,^13,16^ glucose oxidase,^17^ green fluorescent protein,^18^ and lipase.^19,20^ While effective with all of these enzymes, this strategy does require the direct conjugation of monomers to surface residues of the protein, which can lead to a reduction in enzyme activity and even denaturation. To address these challenges, several approaches for noncovalent formation of ENGs have been developed. Gu et al.^21^ published a strategy wherein positively charged monomers are localized around a negatively charged protein. Subsequent polymerization yielded nanogels without the need for covalent modification.^22−24^ Beloqui et al.^25^ demonstrated an effective one-pot synthesis of noncovalent protein nanogels through the addition of sugar excipients such as sucrose. While such noncovalent methods are effective, they rely on intramolecular/electrostatic interactions which inherently limit the pH range, proteins, or monomers that can be used in nanogel formation.

**Figure 1:**
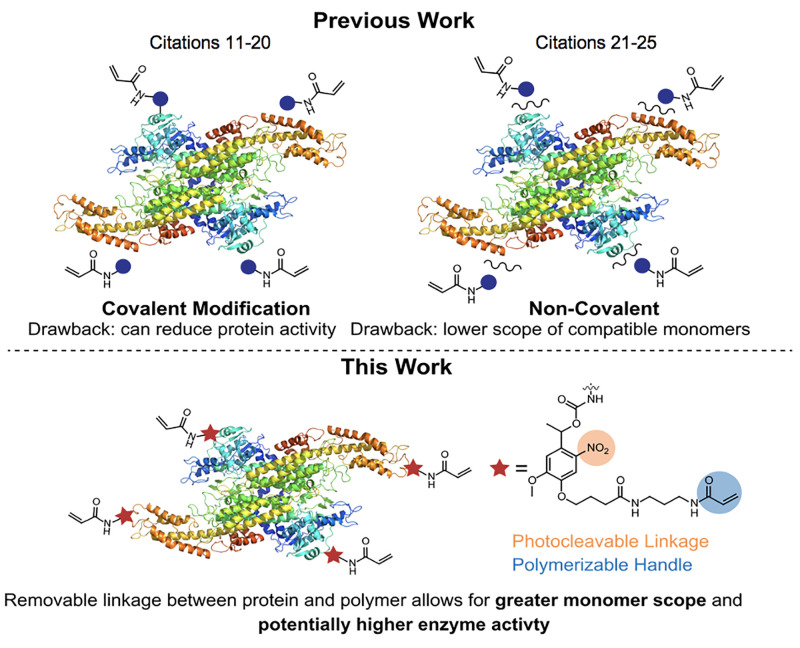
Comparison of strategies for the synthesis of ENGs.

In this work, we present a method to form protein nanogels using a photocleavable monomer that temporarily takes advantages of covalent modification but ultimately allows the enzyme in the final ENG to be noncovalently bound. Conjugation of a bifunctional acrylamide-nitrobenzyl-carbonate (ANC) to lysine residues yields a polymerization handle on the protein surface linked through a photocleavable carbamate (Figure 1). Subsequent polymerization and photoirradiation with 365 nm produce the final noncovalent ENG.

To demonstrate the relevance and utility of this method, we encapsulated phenylalanine ammonia lyase (PAL), an enzyme used in the treatment of phenylketonuria. This disease is characterized by the inability to process dietary phenylalanine, leading to an accumulation of the amino acid and resulting in cognitive impairment.^26^ PAL has been shown to enzymatically transform phenylalanine into the natural metabolites *trans*-cinnamic acid and urea.^27^ Studies have recently led to the development and FDA approval of a PEGylated variant of PAL, which is administered subcutaneously. While this drug is an excellent alternative to current treatments, its reliance on injection is inconvenient to patients. Further, this formulation presents added risk because it is bacterially derived and therefore can illicit an immune response.^28,29^ Thus, a promising, alternative delivery of PAL is oral administration. However, the uniquely harsh environment of the digestive tract requires resistance to degradation at high concentrations of promiscuous proteases and low pH. Reported efforts to address these challenges include genetically engineered PAL via directed evolution^30^ and encapsulation/adsorption of the enzyme onto membranes or other materials.^31−33^ PAL also loses much of its activity when modified at its lysine residues.^34^ As such, PAL represents a relevant model system to study because noncovalent encapsulation is required to maintain activity. Herein, we report the synthesis of non-covalently incorporated PAL nanogels with increased stability against the protease trypsin and low pH compared to the unencapsulated enzyme.

## Results and Discussion

### Design and Synthesis of Photocleavable Acrylamide

**Scheme 1:**
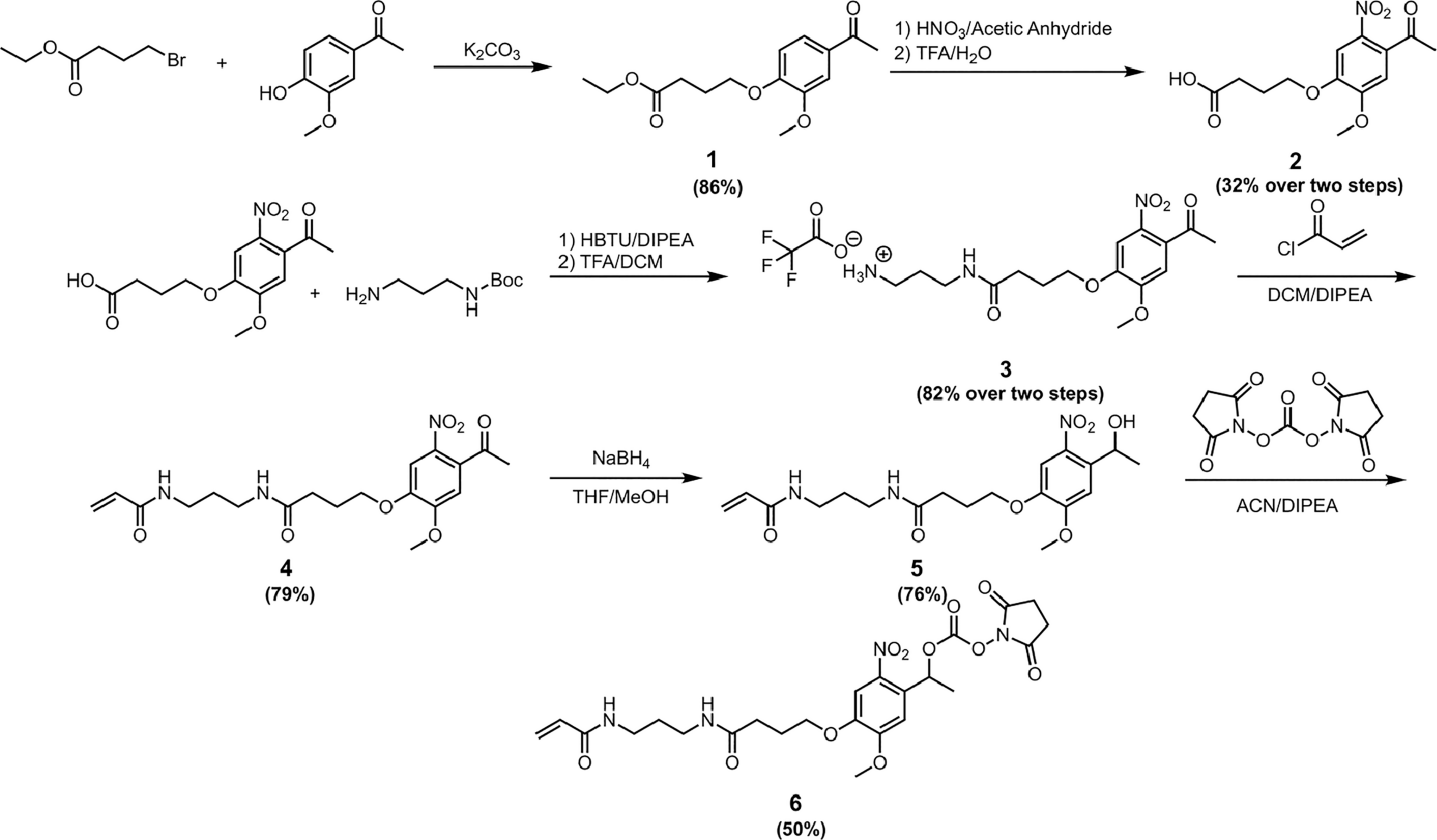
Synthesis of Photocleavable Acrylamide for Templated Nanogels

To facilitate post-polymerization removal of a covalent linkage, an *ortho*-nitro carbamate was used to provide a stable linkage throughout the polymerization process while offering a rapid and traceless mechanism for cleavage using UV light.^35^ Synthesis of the heterobifunctional ANC linked started with acetovanillone (Scheme 1), which could be purchased cheaply at less than $1 USD per gram. Alkylation of the phenol and *ortho*-nitration were performed as previously reported to yield compound **2**.^36^ Coupling and deprotection of *N*-Boc-1,3-propanediamine to **2** yielded a free amine, which was used to install the acrylamide polymerization handle. Subsequent reduction of the ketone using sodium borohydride followed by alkylation with *N*,*N*-disuccinimidyl carbonate resulted in the final lysine-reactive ANC monomer (**6**) able to react with the lysine residues on PAL.

### Expression and Modification of PAL

The PAL variant was recombinantly expressed from *Rhodosporidium toruloides* (Rt) in *Escherichia coli.* This PAL was chosen because it had been previously used in PEGylation studies.^37^ Employing the Rt-PAL gene (GeneBank accession no. X51513), codon optimization was performed. The optimization was important because the protein is native to yeast (optimized DNA sequence: Figure S2). Additionally, a cleavable His_6_-TEV affinity-tag sequence was installed at the N-terminus of the protein to allow for Ni-NTA purification. The optimized sequence was ordered through Twist Biosciences as a pre-cloned expression vector (Ndel_Xhol restriction sites in a pET-29b(+) vector) and was transformed into chemically competent BL21 (DE3) *E. coli*. A small-scale expression of the protein showed a strong induction band after the addition of isopropyl β-d-1-thiogalactopyranoside, but the protein was exclusively in the insoluble fraction after lysis and centrifugation. Therefore, to improve protein solubility, the expression temperature was lowered from 37 to 18 °C, resulting in soluble protein. Purification by a Ni-NTA column followed by the His_6_ cleavage gave the active protein in good yield (approximately 70 mg/L).

In order to provide the maximal number of polymer crosslinking sites on the protein, PAL was highly modified with the ANC linker. Specifically, **6** was added to PAL and excess linker was removed, resulting in a lysine modification of 93% (108 of 116 lysine residues) as measured by the fluorescamine assay. Photocleavage of the linker from the protein, as well as the recovery of protein after UV irradiation, was confirmed by LCMS (Figure S5).

### Synthesis and Characterization of Nanogels

**Figure 2:**
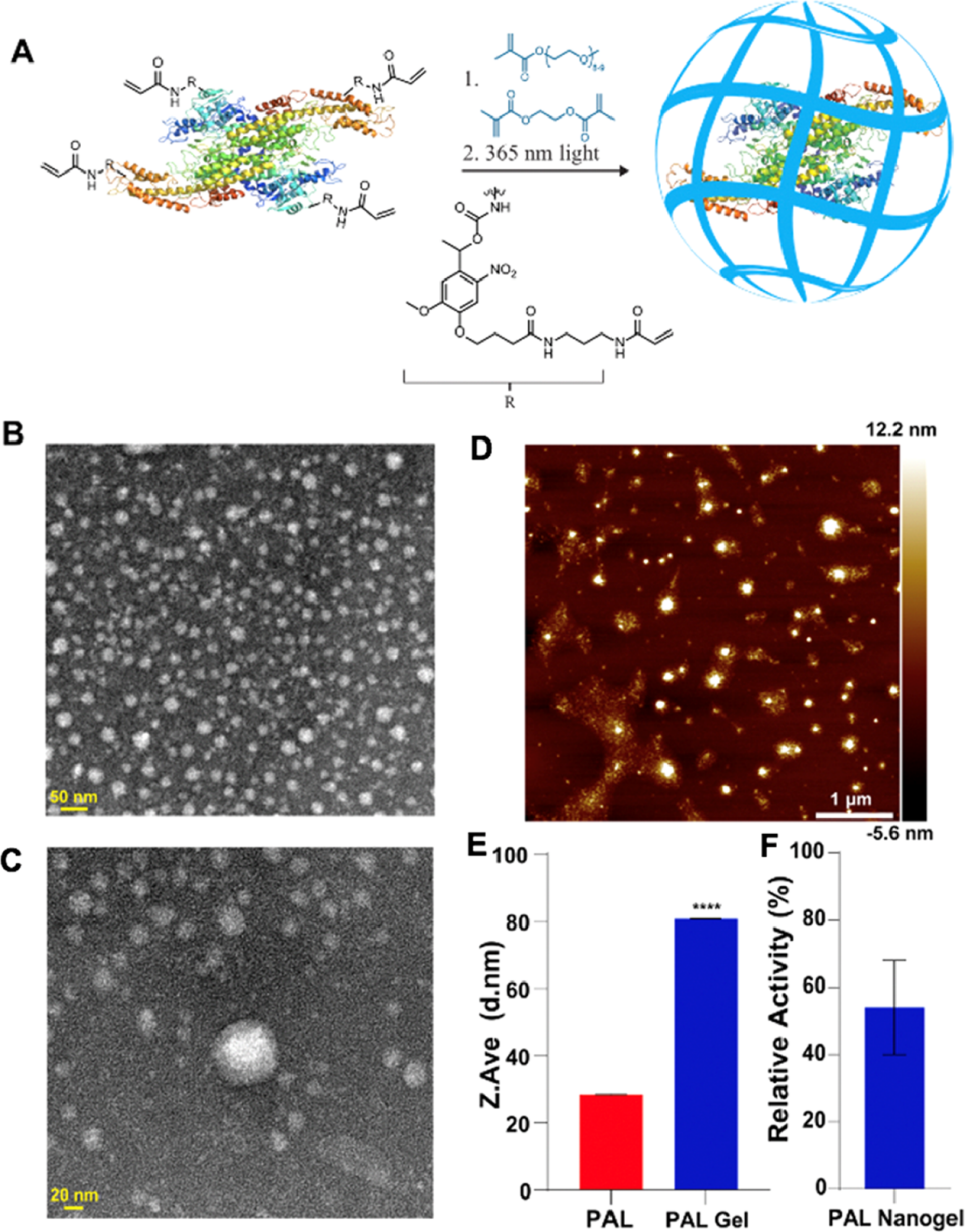
Synthesis of noncovalent PAL nanogels. (A) Scheme for synthesis and photocleavage. Although the figure shows one PAL enzyme in the nanogel for simplicity, it is noted that multiple enzymes could possibly be encapsulated into a single nanogel. The synthesized nanogels post-irradiation were characterized by (B,C) TEM, (D) AFM, and (E) DLS. The activity of the nanogels (F) was measured via HPLC (*n* = 10).

Nanogel synthesis proceeded via polymerization followed by photocleavage (Figure 2A). Poly(ethylene glycol) methacrylate (PEGMA) was chosen as the monomer because of the known biocompatibility of the resulting polymer and its reported ability to protect proteins from protease degradation.^38,39^ Free radical polymerization of PEGMA with ethylene glycol dimethacrylate (EGDMA) as a crosslinker was initiated by an ammonium persulfate (APS)/tetramethylethylenediamine initiator system. Complete incorporation of the acrylamide-modified PAL was confirmed by sodium dodecyl sulfate-polyacrylamide gel electrophoresis based on the absence of an unmodified protein band and a large molecular weight smear for the nanogels (Figure S6). Further validation of the polymerization was conducted via nuclear magnetic resonance (^1^H NMR) wherein clearly identifiable peaks that correlate with the expected polymer backbone and side chains were observed (Figure S7). Fourier transform infrared analysis of the native enzyme and ENG confirmed the formation of the polymer by the appearance of a strong C–O–C stretch at 1079 cm^–1^ of the oligo(ethylene glycol) side chains and a C=O stretch at 1725 cm^–1^ (Figure S8) after polymerization. As expected, peaks distinct to the protein, for example, the C=O stretch at 1632 cm^–1^, were also observed in the gel. The resulting nanogels were purified with spin desalting columns prior to analysis. The morphology of the synthesized nanogels appears to be principally spherical via transmission electron microscopy (TEM, Figure 2B). Cross-sectional analysis of 70 independent particles by atomic force microscopy (AFM, Figure 2C) determined the particle size to be 76 nm (σ = 41 nm). Dynamic light scattering (DLS) showed an increase in size from a *Z*-average of 28 nm (unmodified PAL) to 80 nm (nanogel) with PDIs of 0.53 and 0.46, respectively (Figure 2D; see Figure S9 for DLS intensity, volume, and number percentage data), in agreement with size determination via AFM. It should be noted that both DLS and AFM characterization revealed larger clusters that likely include multiple enzymes within a single nanogel.

As mentioned previously, following the polymerization, the nanogels were irradiated with 365 nm light for 15 min to remove the covalent linkage between the polymer and the enzyme. UV–vis analysis indicated that absorbance of the nanogel at this wavelength is negligible and would therefore not affect the photocleavage reaction (Figure S10). Given that it is possible that more than one PAL is encapsulated in a single gel, it was not possible to quantify the number of amines that were revealed upon exposure to 365 nm light. However, a fluorescamine assay was conducted to demonstrate a relative increase in fluorescence after irradiation of the PAL nanogels with UV light compared to that before irradiation, with UV light suggesting an expected recovery of lysine resides on the protein (Figure S11). Fluorescamine is a reagent that interacts with the primary amino groups of proteins to yield highly fluorescent derivatives.^40^ Thus, we utilized this assay to qualitatively observe that functional amines increased after irradiation. The activity of the PAL in the nanogels was investigated by measuring change in absorbance at 290 nm after incubating with 4 mM phenylalanine for 2 h at 37 °C as adapted from McCallum and Walker.^41,42^ It was not necessary to extend the length of the activity assay due to the enzyme reaching its steady state (Figure S12). The activity of the resulting noncovalent nanogels was 54 ± 14% compared to that of the unencapsulated PAL (Figure 2E). The activity of the nanogels before irradiation was considered negligible since no *trans*-cinnamic acid was detected.

**Figure 3:**
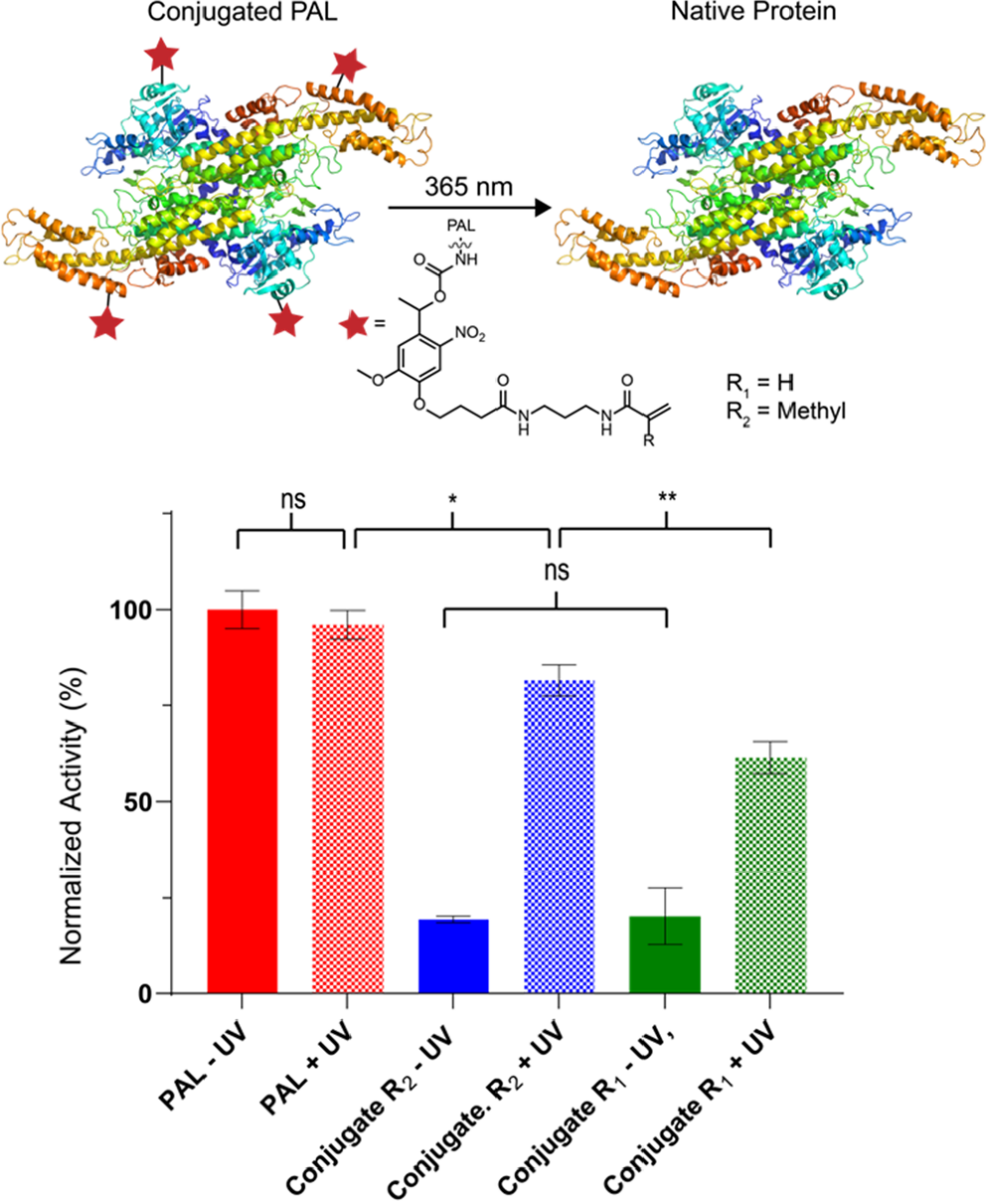
Photocleavage activity model study showing the recovery of enzyme activity after cleavage. Activity measured as an increase in absorbance at 290 nm over the course of 90 min. Each measurement was conducted in triplicate and statistical significance determined via a one-way ANOVA with multiple comparisons (* = *p* ≤ 0.05, ** = *p* ≤ 0.01, **** = *p* ≤ 00001 compared against the unheated sample in each set).

We next attempted to further improve the activity of the PAL in the nanogels. A potential suppressor of enzymatic activity could be irreversible protein modification by the acrylamide on the ANC linker, causing protein modification or crosslinking.^43^ While lysine residues are known to have low reactivity toward acrylamides, it was possible that some cysteine residues in the PAL structure were irreversibly modified through a Michael-addition mechanism, inhibiting enzyme activity. Structurally, PAL contains two unpaired cysteine residues, with one being in a solvent-exposed loop region, giving credence to this hypothesis. To test this, a methacrylamide monomer was synthesized that would have lower reactivity toward cysteine residues. Both the acrylamide and methacrylamide ANC linkers were conjugated to PAL, and the activity was investigated before and after photocleavage with 365 nm light (Figure 3). After conjugating the ANC monomers to the lysine residues of PAL to form the resulting carbamate, a 79.8 ± 9.9% loss in activity was observed for the acrylamide (R_1_ in Figure 3) and an 80.7 ± 4.2% loss for the methacrylamide (R_2_ in Figure 3) compared to fresh protein. After exposing the acrylamide conjugate to 365 nm light, the enzyme regained 62.9 ± 9.7% of its activity while the methacrylamide regained 81 ± 4% original activity, thus supporting the fact that methacrylamide recovers higher activity than the acrylamide linker. Irradiation with 365 nm light (PAL + UV in Figure 3) did not have a statistically significant effect on PAL activity, which is encouraging as UV light is known to damage some proteins.^44^ The results demonstrate that the activity of the protein is deleteriously affected by modifying the protein at the lysine residues and further exemplifies the need to have a removable linkage in the nanogel design. Interestingly, the data also showed a statistically significant difference in enzymatic activity after photocleavage between the acrylamide and methacrylamide (18.1 ± 10.5% difference). This result does support the hypothesis that the acrylamide of the ANC linker modifies the PAL irreversibly, although further studies will need to be undertaken to definitively test this. While the methacrylamide conjugate recovered higher activity once irradiated, PAL nanogels could not be prepared with this conjugate using the same polymerization conditions. This was likely a result of the different reactivity of methacrylamide compared to that of acrylamide.^45^ In future studies, further optimization will need to be undertaken to determine the conditions necessary to synthesize ENGs using the methacrylamide linker.

### Stabilization of Nanogels to Trypsin and pH

**Figure 4:**
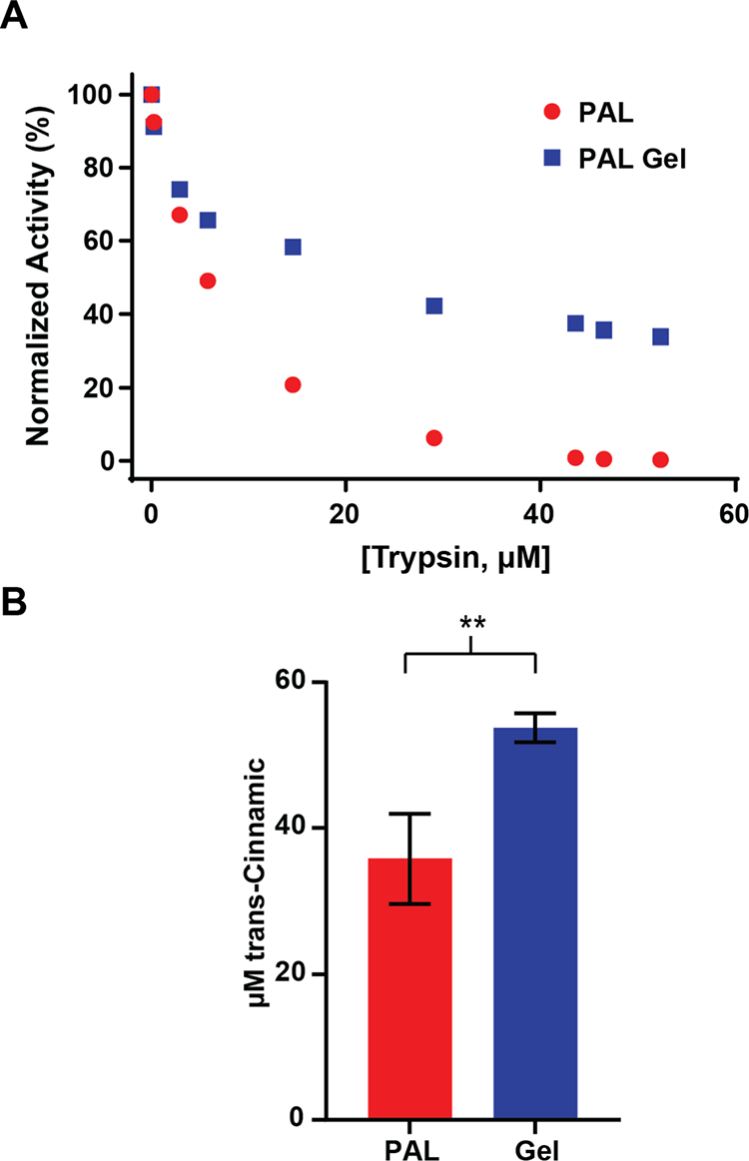
(A) Normalized dose response stabilization of nanogels against trypsin as measured by an increase in A_290_. Each blue data point represents an individually synthesized batch of nanogel. B) Total production of *trans*-cinnamic acid produced after 2 h in the presence of 7.5 μM trypsin. Concentration determination was determined via HPLC (*n* = 3).

PEG nanogels are known to protect proteins from proteases.^46^ In order to test this, the PAL nanogels were exposed to a range of trypsin concentrations that encompass the reported protease levels in the small intestine (0–40 μM).^47^ Activity was measured by monitoring the increase in absorbance at 290 nm after 2 h of incubation; this corresponds to the concentration of *trans*-cinnamic acid and thus enzymatic activity. In this assay (Figure 4a), the PEG nanogel stabilizes PAL, as the activity is higher than PAL alone for trypsin concentrations equal to and above 0.25 μM. Furthermore, the nanogel maintained 34% of its original activity at a trypsin concentration of 52 μM, which is above the reported physiological concentration of trypsin. In contrast, unmodified PAL shows no measurable activity under identical conditions. Due to time-consuming nature of these experiments (where each single point represented a newly synthesized nanogel), this dose-response study could not feasibly be done in replicates. To compensate and verify results, we measured the PAL gel versus PAL activity at a single trypsin concentration against a *trans*-cinnamic acid standard via high-performance liquid chromatography (HPLC). In this assay, the nanogel achieved a statistically significant improvement in total enzymatic output over the 2 h exposure to 7.5 μM trypsin (Figure 4b).

The crosslinking density of the nanogel was then altered to determine its effect on protein stabilization to trypsin. We initially hypothesized that a greater molar equivalence ratio of crosslinker to monomer would increase the stability of PAL to trypsin as it would be more sterically protected from being degraded by the protease. To test this, PAL nanogels were synthesized with a range of crosslinking ratios starting from a 48:0 monomer to crosslinker molar equivalence ratio to a 48:36 monomer to crosslinker molar equivalence ratio. The total concentration of monomer and crosslinker was maintained to allow for insignificant changes in polymerization kinetics. All nanogels were exposed to 6 μM trypsin, and activity was measured as done previously. As predicted, zero crosslinking density did not protect the protein from trypsin degradation (Figure S13). Stability was positively correlated with an increase in crosslinking molar equivalences until a 48:10 monomer to crosslinker ratio. Higher crosslinking density seemed to have no significant increase in protein stability, although further studies can be conducted to optimize the system toward other proteases such as pepsin.

**Figure 5:**
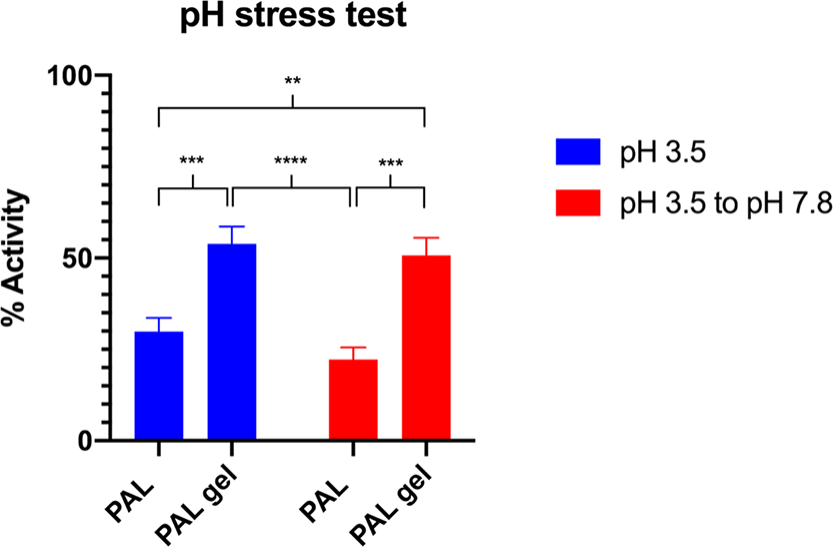
Normalized stabilization of nanogels against low pH of 3.5 measured by an increase in A_290_. The activities of the PAL samples were measured via a plate reader assay. Each measurement was conducted in triplicate, and statistical significance was determined via a two-way ANOVA with multiple comparisons (** = *p* ≤ 0.001, *** = *p* ≤ 0.0005, **** = *p* < 0.0001). Normalization was done by comparing all samples against an unmodified PAL control measuring activity at pH 7.8.

Another major obstacle in the delivery of therapeutic enzymes by oral administration is the harsh conditions of the low pH in the stomach. The acidic environment can destabilize the protein due to hydrolysis. To test the ability of the nanogel to stabilize the protein, PAL nanogels were first buffer exchanged into a pH 3.5 buffer to mimic the stomach fluid (during eating) before introduction of phenylalanine to initiate the activity assay. After 0.5 h from the addition of phenylalanine, the nanogels were either maintained at pH 3.5 or allowed to be brought up to pH 7.8, which is the optimal pH for PAL activity. Activity was measured as described above monitoring an increase in absorbance at 290 nm. In this assay (Figure 5), the PAL nanogels subjected to a low pH maintained a higher activity percentage than PAL alone subjected to the same pH of 3.5. This result supports that the nanogel stabilized the microenvironment for the enzyme, thereby increasing its tolerance to low pH.

## Conclusions

Encapsulation of enzymes in nanogels is an excellent method to protect and deliver proteins for the treatment of metabolic diseases. Herein, we report a new strategy for synthesizing noncovalent ENGs that rely on a bifunctional ANC linkage between the lysine residues of the enzyme and polymer. PEGMA was polymerized in the presence of the modified PAL enzyme and a crosslinker to form nanogels as determined by DLS, AFM, and TEM. Subsequent UV irradiation liberated PAL from covalent immobilization, allowing for enhanced enzyme activity.

The synthesized nanogels confer PAL the ability to resist trypsin and low pH degradation, indicating the potential utility of these PAL ENGs for oral administration. Since phenylalanine is a nonessential amino acid and is thus only introduced to the body by food, delivery of PAL ENGs could be useful by an oral route, taken at the same time as meal consumption. These ENGs may be further tuned to meet desired properties depending on the application. For example, other monomer systems such as *N*-isopropylacrylamide-*co*-acrylic acid could be used to prepare a nanogel that can control swelling of gel according to pH, temperature, or other stimuli. Furthermore, this proof-of-concept work could be potentially employed with a variety of other therapeutic enzymes for protein delivery applications.

## Methods

### Expression of PAL from Rt

Plasmid was transformed into chemically competent BL21 (DE3) *E. coli,* and starter cultures were grown in terrific broth (TB) with 50 μg/mL kanamycin. The following day, these starter cultures were used to inoculate 2 × 750 mL shaker flasks of TB broth (containing 50 μg/mL kanamycin). Cells were grown at 37 °C until a OD_600_ of 1.3 was reached. Cells were then induced with 1 mM IPTG, and expression was allowed to occur for 16 h at 18 °C. After expression, the cells were pelleted via centrifugation and resuspended in lysis buffer (50 mM Tris, pH 7.8, 300 mM NaCl, 10 mM imidazole, one Roche cOmplete protease inhibitor tablet). The cells were then lysed with an emulsifier, and the supernatant was clarified via centrifugation. The supernatant was then incubated with Ni-NTA resin and rocked at 4 °C for 15 min. The resin slurry was then poured into a gravity column, and the flowthrough was collected. The bound protein was then washed with 200 mL of wash buffer (50 mM Tris, pH 7.8, 300 mM NaCl, 10 mM imidazole). The bound His-tagged protein was then eluted with 50 mL of elution buffer (50 mM Tris, pH 7.8, 300 mM NaCl, 300 mM imidazole) and then dialyzed into 50 mM Tris, pH 7.8, 300 mM NaCl for 16 h.

For removal of the His-tag, the His_6_-TEV-PAL was incubated with 1:10 weight equivalents of TEV protease and allowed to incubate for 9 h at 4 °C. To the mixture, Ni-NTA resin was added, and the slurry was rocked at 4 °C for 15 min. The slurry was added to a gravity column, and the flowthrough was collected. The unbound protein was then eluted with 50 mL of wash buffer (50 mM Tris, pH 7.8, 300 mM NaCl, 10 mM imidazole). The TEV-cleaved protein was then dialyzed into PAL buffer (50 mM Tris, pH 7.8, 90 mM NaCl) for 16 h, and the protein was stored at −80 °C until use.

### Conjugation

A 4 mg/mL solution of expressed PAL (stored at −80 °C) was thawed for 30 min at 4 °C. The protein was then buffer exchanged into pH 9, 50 mM sodium carbonate buffer via a 3 kDa MWCO Centriprep filter. After the protein concentration was determined via a BCA assay, a photocleavable linker (1.64 mg, 2.9 μmol, 4 equiv with respect to lysine residues) dissolved in dimethyl sulfoxide (DMSO) was added (final DMSO concentration of 5%). The conjugation reaction was allowed to proceed for 5 h at 25 °C. Excess linker was removed via 7 kDa MWCO Zeba desalting spin columns, and the modified PAL was buffer exchanged into PAL buffer via 3 kDa MWCO Centriprep filters.

### Photocleavage Protocol

Samples were irradiated with UV light (365 nm) for 15 min at 4 °C to allow for photocleavage of the linker. Afterward, the samples were passed through a 7 kDa MWCO Zeba desalting spin column to remove excess monomer, photocleavage byproducts, and other small molecules. The desalting column was initially equilibrated with PAL buffer.

### Fluorescamine Assay

Briefly, 100 μL of nanoparticle PAL (or modified PAL solution) was added to a 96 opaque well plate along with 30 μL of a 3 mg/mL fluorescamine solution (prepared in DMSO). The plate was incubated at 37 °C for 30 min. Fluorescence was then measured at an excitation wavelength of 380 nm and an emission wavelength of 460 nm.

### Recovery of PAL Activity after Photocleavage

To determine percent recovery after photocleavage, each of the conjugated samples was compared against unmodified PAL. Conjugated samples underwent UV irradiation (365 nm) for 15 min at 4 °C as described previously. Afterward, the samples were passed through a 7 kDa MWCO Zeba desalting spin column to remove photocleavage byproducts and other small molecules. The desalting column was equilibrated with PAL buffer. The remaining protein solutions were added to a 96-well plate and diluted to 200 μL with PAL buffer. The assay was initiated through the addition of 30 μL of 4 mM phenylalanine in PAL buffer, and kinetics were monitored by measuring absorbance values at 290 nm every 30 s for 2 h at 37 °C. Enzyme activity was determined by calculating the difference in absorbance values between the first and last time points. These values were normalized to that of the unmodified PAL.

### Nanogel Synthesis

PEGMA_500_ (20 μL, 0.05 mmol, 50 equiv), EGDMA (2 μL, 0.01 mmol, 10 equiv), tetramethylethylenediamine (0.6 μL, 0.004 mmol, 4 equiv), and conjugated PAL (12 μg) were added to a 4 mL dram vial with a stir bar and diluted up to 0.875 mL with PAL buffer. The mixture was allowed to degas under argon for 45–60 min. In a separate dram vial, APS stock solution in PAL buffer was prepared and also degassed under argon for 45–60 min. After degassing, 0.125 mL (0.2 mg, 1 μmol, 1 equiv) of the APS solution was added to the reaction mixture, and the polymerization was allowed to proceed for 2 h. To quench the reaction, samples were exposed to air.

### Dose-Response Trypsin Challenge Assay

Ten individual PAL nanogels were prepared as described previously. For photocleavage, the gels were transferred to individual wells of a 48-well culture plate. The portion of the plate holding the samples was then irradiated with UV light (365 nm) for 15 min at 4 °C to allow for photocleavage of the linker. Afterward, the samples were passed through 7 kDa MWCO Zeba desalting spin columns to remove photocleavage byproducts and other small molecules. Buffer containing 50 mM Tris and 90 mM NaCl (pH 7.8) was used as equilibration buffer during this small-molecule cleanup.

The purified gel solutions were next split in half and added to individual wells on a UV transparent 96-well plate. One-half of the gel solution (20 μL) was added to a well consisting of a given concentration of trypsin (see below^*a*^), 95–195 μL PAL buffer, and 30 μL of 1% TFA 50/50 MeOH/ACN to immediately quench catalytic activity (these samples serve as *t* = 0 h). The second half of the gel solution was added to a well consisting of the same given concentration of trypsin as the first half of the gel solution in PAL buffer. All samples had a total volume of 275 μL. Lastly, 30 μL of 4 mM phenylalanine in 50 mM Tris base, 90 mM NaCl pH 7.8 buffer was added to all wells to initiate activity.

Absorbance measurements at 290 nm (37 °C) were taken every 30 s for 2 h. The catalytic activity was measured by calculating the difference between the absorbance measurements at *t* = 2 h and *t* = 0 h for each sample.

^*a*^The following concentrations of trypsin were used for this experiment: 0, 0.25, 2.9, 5.8, 14.5, 29.1, 43.6, 46.5, 52.4, and 58.2 μM.&quot; to here (after the Dose-Response Trypsin Challenge Assay.

### Single Point HPLC Trypsin Challenge Assay

Six individual nanogels were prepared and photocleaved as described previously. Next, 150 μL of a 70 μM trypsin solution was added to the unencapsulated PAL and PAL nanogels (1200 μL) in 1.5 mL LoBind Eppendorf tubes. Lastly, 100 μL of 4 mM phenylalanine in PAL buffer was added to initiate the activity assay. The tubes were allowed to incubate in a thermoshaker for 2 h at (37 °C and 250 rpm). Afterward, samples were then passed through Centriprep 3 kDa MWCO filters to collect *trans*-cinnamic acid. Samples were filtered, and *trans*-cinnamic acid concentration was determined against a standard curve of a pure reference.

### Low pH Assay

Six individual nanogels were prepared and photocleaved as described previously. Next, samples of unencapsulated PAL and PAL nanogels were buffer exchanged into a pH 3.5 citric acid buffer via Centriprep 30 kDa MWCO filters. The resulting volumes of the samples were 120 μL and added to individual wells on a UV transparent 96-well plate. Then, 30 μL of 4 mM phenylalanine in PAL buffer was added to each well to initiate the activity assay. Absorbance measurements at 290 nm (37 °C) were taken every 30 s for 2 h. At the 1 h mark, samples were either maintained at pH 3.5 or brought up to pH 7.8. The catalytic activity was measured by calculating the difference between the absorbance measurements at *t* = 2 h and *t* = 0 h for each sample. Normalization was done by comparing all samples against an unmodified PAL control measuring activity at pH 7.8.

## Supporting Information Available

Experimental details for the synthesis of monomer, materials, analytical techniques, characterization of protein, ^1^H and ^13^C NMR spectra, and additional figures (PDF)
