# Supplementary material for: Noncovalent Enzyme Nanogels via a Photocleavable Linkage
Source: Macromolecules. 2022 Nov 3;55(22):9925–33. doi: 10.1021/acs.macromol.2c01334 (PMC9686129; doi:10.1021/acs.macromol.2c01334)
Supplement: Supplementary file 1 — ma2c01334_si_001.pdf [file ma2c01334_si_001.pdf]

Noncovalent Enzyme Nanogels via a Photocleavable Linkage

‡Neil L. Forsythe,[1] ‡Mikayla Tan,[1] Daniele Vinciguerra,[1] Jacquelin Woodford,[1] Adam Z. Stieg,[2] and Heather D. Maynard*[1,2]

[1]Department of Chemistry and Biochemistry, 607 Charles E. Young Drive East, University of California, Los Angeles, California 90095.
[2]California NanoSystems Institute, 570 Westwood Plaza Building 114, University of California, Los Angeles, California, 90095.
‡NLF and MST Contributed Equally
*Corresponding author, Email: maynard@chem.ucla.edu

Supporting Information – Table of Contents

Materials and Methods. 2
Analytical Techniques 2
Photocleavable Monomer Synthesis. 3
Figure S1. pET-29b Plasmid map for the expression of Phenylalanine Ammonia Lyase 7
Figure S2 cDNA used for Rt-PAL expression 8
Figure S3. SDS PAGE of His-cleaved PAL product. 9
Figure S4. Deconvoluted mass spectrometry of His-cleaved PAL product. 9
Figures S5. LCMS PAL, ANC-modified PAL, and the crude product of the photocleavage. 10
Figure S6. SDS-PAGE of unencapsulated PAL and purified nanogels 11
Figure S7. ^{1}H NMR of PAL nanogels 12
Figure S8. Fourier transform infrared spectrum of PAL and PAL nanogels. 12
Figure S9. Dynamic Light Scattering of PAL and PAL nanogels 13
Figure S10. UV-Vis Spectrum of PAL nanogels. 14
Figure S11. Fluorescamine assay of PAL nanogels 14
Figure S12. PAL activity kinetics 15
Figure S13. Varying crosslinking density 16
Figure S14. Plate reader assay control experiment 17
References 17
^{1}H and ^{13}C NMR 18

Materials and Methods.

Acetovanillone was purchased form Oakwood Chemicals. All other chemicals were purchased from Sigma-Aldrich and Fisher Scientific and were used without purification unless otherwise noted. For PEGMA, inhibitor was removed by passing through a plug of basic alumina. Plasmid for PAL expression was ordered from Twist Biosciences with the sequence cloned between NdeI_XhoI restriction sites in a pET-29b(+) vector. TEV protease was expressed following reported procedures and stored at -80 °C in 50% glycerol and thawed immediately before use.[1] For photocleavage experiments, a 4 watt, 365 nm lamp was used (brand: UVP).

Analytical Techniques. Nuclear Magnetic Resonance (NMR) spectra were recorded on a Bruker AV 400 MHz, Bruker DRX 500 MHz, Bruker AV 500 MHz, and Bruker AV 600 MHz spectrometer. Proton NMR spectra were acquired with a relaxation delay of 2 s for small molecules and 10 seconds for polymers. Abbreviations are s=singlet, d=doublet, t=triplet, q=quartet, p=pentet, dd=doublet of doublets. Mass spectrometry for both proteins and small molecules was obtained on an Agilent Q-TOF 6530 LC/MS using a 10-95% acetonitrile gradient over 15 minutes. For SDS PAGE, BioRad Any kD Mini-PROTEAN-TGX™ gels were used. SDS-PAGE protein standards were obtained from Bio-Rad (Precision Plus Protein Prestained Standards). Small molecule purification was done via flash chromatography and conducted on a Biotage Isolera One auto-column system. Atomic force microscopy (AFM) images were acquired in the PeakForce Tapping mode on a Nanoscope V Dimension Icon instrument (Bruker AXS) under ambient conditions using a silicon tip silicon on a nitride cantilever (ScanAsyst-Air-HPI, Bruker) with a nominal spring constant of 0.25 N/m, first longitudinal resonance frequencies between 35 and 75 kHz, and a nominal tip radius of 2 nm. Topographic images were acquired and reproduced from multiple samples. Simple first-order plane fitting of the acquired images enabled subsequent cross-sectional analyses. Dynamic light scattering (DLS) measurements were carried out in triplicate using a Malvern Zetasizer Nano (173° scattering angle) at a temperature of 25 °C to determine size (diameter) and polydispersity. The nanogels were prepared at 4 mg/ml and diluted to 0.04 mg/mL in DPBS. Transmission electron microscopy (TEM) images were acquired on a FEI T12 instrument using formvar/carbon coated grids (200 mesh, Cu, Ted Pella). Grids were glow discharged for 35 seconds. 5 μL of sample at 0.04 mg/mL were placed on the grid and allowed to adhere for 3 minutes. After removing excess solution with filter paper, the grids were stained with 4 μL of a 2.5 % uranyl acetate solution for 2 minutes.

Photocleavable Monomer Synthesis.

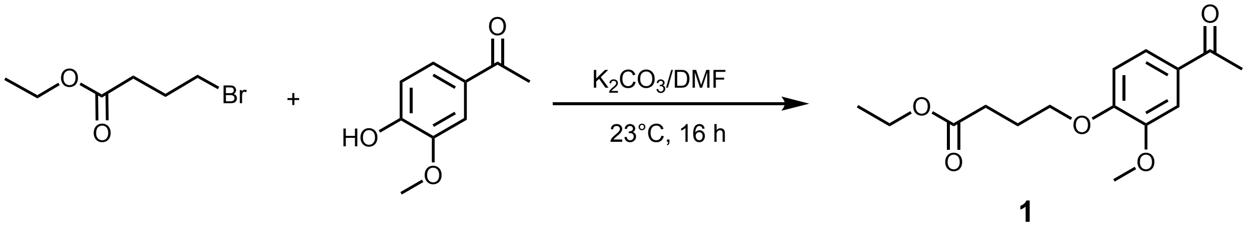

1

(86%)

Synthesis of 1.[2] Acetovanillone (5.0 g, 30 mmol,1 equivalent) and K_2CO_3 (6.2 g, 45 mmol, 1.5 equivalents) were dissolved in 40 mL of DMF. Ethyl 4-bromobutyrate (5.2 mL, 36 mmol, 1.2 equivalents) was added dropwise and the reaction was allowed to proceed for 12 h. The crude was then poured into 400 mL of cold water and the resulting solid was collected via filtration, washed with excess water, and dried to yield 7.28 g (86%) of a brown powder. ^{1}H NMR (600 MHz, chloroform-*d*) δ 7.55 (dd, *J* = 8.3, 2.0 Hz, 1H), 7.52 (d, *J* = 2.0 Hz, 1H), 6.89 (d, *J* = 8.4 Hz, 1H), 4.14 (q, *J* = 6.6, 6.0 Hz, 4H), 3.91 (s, 3H), 2.56 (s, 3H), 2.54 (t, *J* = 7.2 Hz, 2H), 2.19 (p, *J* = 6.8 Hz, 2H), 1.25 (t, *J* = 7.1 Hz, 3H). ^{13}C NMR (101 MHz, chloroform-*d*) δ 196.90, 173.08, 152.67, 149.30, 130.53, 123.24, 111.29, 110.50, 67.83, 60.54, 56.02, 30.61, 26.23, 24.32, 14.23. IR: ν = 2613, 2940, 1730, 1672, 1587, 1509, 1466, 1416, 1356, 1266, 1218, 1176, 1149, 1134, 1027, 876, 807 cm^{-1}. ESI-MS calculated for $C_{15}H_{20}O_5$ $[M+H]^+$ = 281.1389, observed = 281.1391.

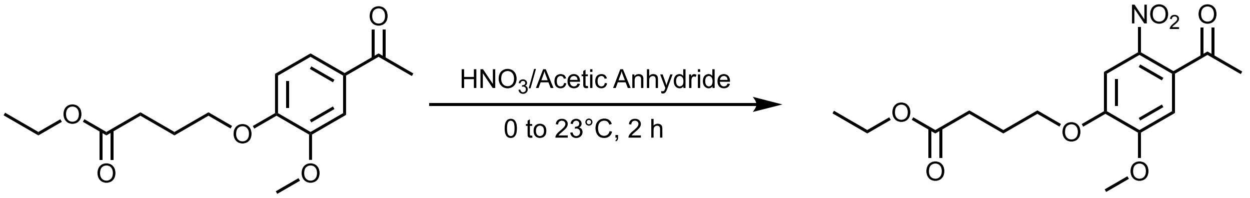

(41%)

Nitration. Alkylated acetovanillone **1** (7.28 g, 26 mmol, 1 equivalent) was dissolved in acetic anhydride (30 mL, 320 mmol, 12 equivalents) and added dropwise to 70 mL of 70% nitric acid at 0 °C. After 30 minutes, the reaction was warmed to room temperature and allowed to proceed for 1.5 h. The mixture was them precipitated into 400 mL of cold water and the resulting solid was collected via filtration. The product was further purified *via* recrystallization from ethanol followed by vacuum filtration to yield 3.50 g (41%) of a yellow powder. ^{1}H NMR (400 MHz, chloroform-*d*) δ 7.61 (s, 1H), 6.74 (s, 1H), 4.22 – 4.11 (m, 4H), 3.95 (s, 3H), 2.54 (t, *J* = 7.2 Hz, 2H), 2.49 (s, 3H), 2.20 (p, *J* = 6.7, 2H), 1.26 (t, *J* = 7.1 Hz, 3H). ^{13}C NMR (101 MHz, chloroform-*d*) δ 200.11, 172.82, 154.32, 148.90, 138.41, 132.88, 108.79, 108.04, 68.51, 60.63, 56.61, 30.53, 30.41, 24.20, 14.24. IR: ν = 2298, 1731, 1708, 1521, 1337, 1284, 1214, 1182, 1039 cm^{-1}. ESI-MS calculated for $C_{15}H_{19}NO_7$ $[M+H]^+$ = 326.1240, observed = 326.1289.

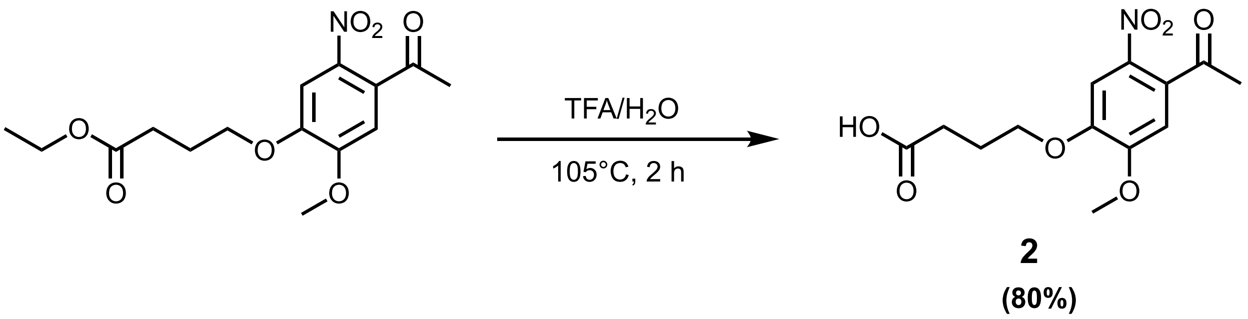

Synthesis of 2. Ethyl protected starting material was added to a round bottom flask and suspended in 50 mL of water along with 20 mL of trifluoroacetic acid. The reaction was heated to 105 °C and stirred for 2 h. The reaction was then cooled and TFA was removed via Hi Vac. The resulting precipitate was collected via vacuum filtration, washed with water, and dried to yield 2.57 g (80%) of a yellow solid. ^{1}H NMR (500 MHz, DMSO-*d*$_6$) δ 12.16 (s, 1H), 7.62 (s, 1H), 7.20 (s, 1H), 4.10 (t, J = 6.5 Hz, 2H), 3.91 (s, 3H), 2.49 (s, 3H), 2.37 (t, J = 7.3 Hz, 2H), 1.94 (p, J = 6.9 Hz, 2H). ^{13}C NMR (126 MHz, DMSO-*d*$_6$) δ 199.77, 174.43, 153.76, 148.97, 138.82, 131.61, 110.31, 108.49, 68.63, 57.14, 30.48, 30.33, 24.36. IR: ν = 2309, 1697, 1518, 1503, 1330, 1281, 1213 cm^{-1}. ESI-MS calculated for $C_{13}H_{15}NO_7$ $[M+H]^+$ = 298.0926, observed = 298.0982.

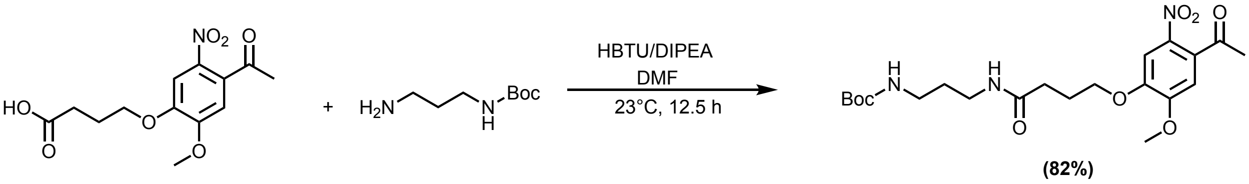

Boc Propyl Amine Coupling. Nitrated acid **2** (1.05 g, 3.53 mmol, 1 equivalent), HBTU (2.01 g, 5.30 mmol, 1.5 equivalents), and *N,N*-diisopropylethylamine (1.54 mL, 8.83 mmol, 2.5 equivalents) were dissolved in 30 mL DMF. The reaction was stirred for 30 min to form the activated complex (forms an insoluble precipitate). N-Boc-1,3-propanediamine (678 µL, 3.89 mmol, 1.1 equivalents) was then added and reaction was allowed to proceed for 12 h. The mixture was then poured into 300 mL of cold water and the precipitate was collected via filtration. The product was then dissolved in DCM, dried with $MgSO_4$, and concentrated to yield 1.32 g (82 %) of a yellow powder. ^{1}H NMR (400 MHz, chloroform-*d*) δ 7.61 (s, 1H), 6.74 (s, 1H), 6.39 (s, 1H), 4.84 (s, 1H), 4.16 (t, J = 6.2 Hz, 2H), 3.96 (s, 3H), 3.30 (q, J = 6.2 Hz, 2H), 3.14 (m, 2H), 2.49 (s, 3H), 2.44 (t, J = 7.2 Hz, 2H), 2.22 (p, J = 6.7 Hz, 2H), 1.61 (p, J = 6.5 Hz, 2H), 1.43 (s, 9H). ^{13}C NMR (126 MHz, chloroform-*d*) δ 200.14, 172.24, 156.75, 154.25, 148.91, 138.43, 132.81, 108.76, 108.08, 79.53, 68.79, 56.61, 35.91, 32.60, 30.42, 30.27, 28.38, 24.76. IR: ν = 3352, 2309, 1736, 1525, 1364, 1287, 1216 cm^{-1}. ESI-MS calculated for $C_{21}H_{31}N_3O_8$ $[M+Na]^+$ = 476.2008 observed = 476.2077.

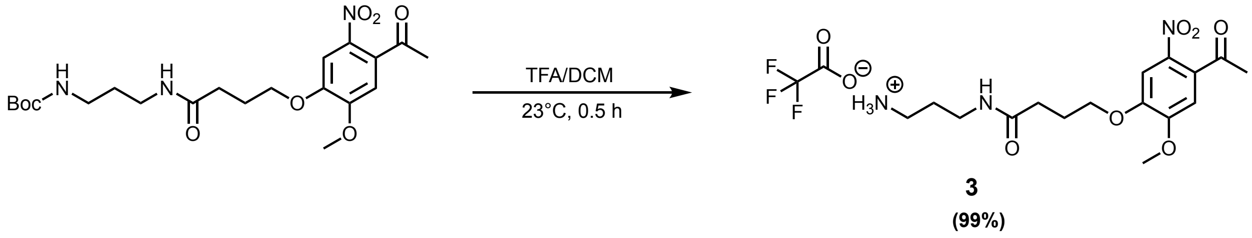

Synthesis of 3. Boc protected starting material was dissolved in 10 mL of 50/50 TFA:DCM in a scintillation vial and stirred for 30 min. TFA was then removed via vacuum and the crude was then dissolved in DCM and precipitated/triturated in 50 mL of diethyl ether to yield the product quantitatively as a yellow wax. ^{1}H NMR (500 MHz, DMSO-d_6) δ 8.03 (t, J = 5.8 Hz, 1H), 7.70 (s, 3H), 7.61 (s, 1H), 7.21 (s, 1H), 4.09 (t, J = 6.4 Hz, 2H), 3.91 (s, 3H), 3.10 (q, J = 6.5 Hz, 2H), 2.81 – 2.71 (m, 2H), 2.53 (s, 1H), 2.50 (s, 3H), 2.24 (t, J = 7.5 Hz, 2H), 1.95 (p, J = 6.7 Hz, 2H), 1.65 (p, J = 7.0 Hz, 2H). ^{13}C NMR (126 MHz, DMSO-d_6) δ 199.78, 172.38, 153.73, 149.00, 138.81, 131.60, 110.30, 108.41, 68.99, 57.13, 37.27, 36.01, 31.90, 30.49, 27.95, 24.98. IR: ν = 2942, 1677, 1645, 1518, 1335, 1282, 1199, 1175, 1132, 1036. ESI-MS calculated for $C_{16}H_{24}N_3O_6$ $[M]^+$ = 354.1665, observed = 354.1828.

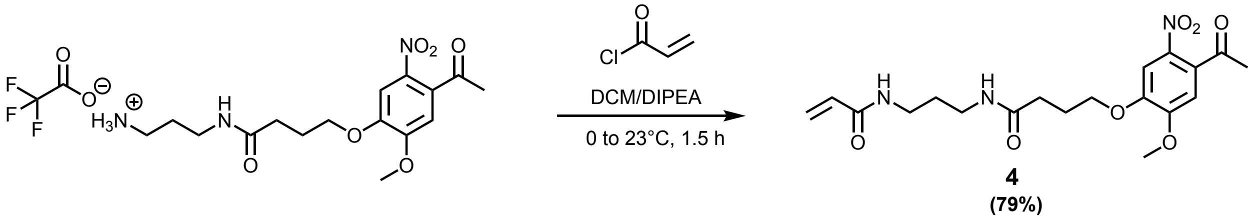

Synthesis of 4. Trifluoroacetate salt **3** (200 mg, 428 μmol) was dissolved in 5 mL of DCM with DIPEA (298 μL, 1.71 mmol, 4 equivalents) and added to a scintillation vial. Reaction was then cooled to 0 °C and acryloyl chloride[1] (52 μL, 642 μmol) was added dropwise. Reaction was stirred for 30 min at 0 °C and then stirred at 22 °C for 1 h. Reaction was then concentrated and the product purified via column chromatography with a 95:5 DCM:MeOH mobile phase (via gradient on autocolumn). 137 mg (79 %) of product was isolated as a yellow powder. ^{1}H NMR (600 MHz, chloroform-d) δ 7.64 (s, 1H), 6.75 (s, 1H), 6.38 (s, 1H), 6.29 (dd, J = 17.0, 1.4 Hz, 2H), 6.13 (dd, J = 17.0, 10.3 Hz, 1H), 5.67 (dd, J = 10.3, 1.4 Hz, 1H), 5.30 (s, 1H), 4.17 (t, J = 6.2 Hz, 2H), 3.97 (s, 3H), 3.36 (q, J = 6.3 Hz, 2H), 3.31 (q, J = 6.2 Hz, 2H), 2.50 (s, 3H), 2.46 (t, J = 7.1 Hz, 2H), 2.23 (p, J = 6.7 Hz, 2H), 1.67 (m, 2H). ^{13}C NMR (126 MHz, chloroform-d) δ 200.14, 172.64, 166.28, 154.26, 148.88, 138.42, 132.85, 130.82, 126.60, 108.75, 108.10, 68.78, 56.63, 35.80, 32.64, 30.42, 29.78, 24.74. IR: ν = 3002, 1737, 1365, 1217. ESI-MS calculated for $C_{19}H_{25}N_3O_7$ $[M+H]^+$ = 408.1771, observed = 408.1875.

[1] For the methacrylamide linker, methacryloyl chloride was added in place of acryloyl chloride. ^{1}H NMR (500 MHz, Chloroform-d) δ 7.63 (s, 1H), 7.06 (s, 1H), 6.65 (s, 1H), 6.49 (q, J = 6.4 Hz, 1H), 6.36 (s, 1H), 5.76 (s, 1H), 5.34 (s, 1H), 4.18 – 4.08 (m, 3H), 4.04 (s, 3H), 3.30 (m, 4H), 2.80 (s, 4H), 2.45 (t, J = 7.1 Hz, 2H), 2.21 (p, J = 6.8 Hz, 2H), 1.99 – 1.97 (s, 3H), 1.76 (d, J = 6.3 Hz, 3H), 1.64 (p, J = 6.1 Hz, 3H).

^{13}C NMR (126 MHz, Chloroform-*d*) δ 172.87, 168.90, 168.48, 154.52, 150.60, 147.68, 139.74, 139.30, 131.22, 119.93, 109.17, 107.33, 76.59, 68.58, 56.54, 35.84, 35.71, 32.89, 29.80, 25.43, 24.87, 21.95, 18.64. Calculated for $C_{25}H_{32}N_4O_{11}$ [M+H] = 565.2146, Observed = 565.2260.

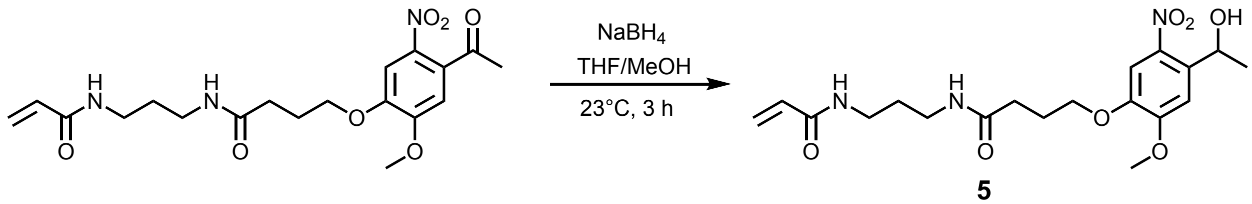

5
(76%)

Synthesis of 5. Ketone acrylamide **4** (158 mg, 388 µmol, 1 equivalent) was dissolved in 5 mL of 3:2 THF/MeOH with a stir bar in a scintillation vial. Sodium borohydride (88 mg, 2.33 mmol, 6 equivalents) was then added in portions, resulting in the evolution of H_2. Reaction was allowed to proceed at room temperature for 3 h. Saturated NH_4Cl was then added to quench and the organic layer was collected. The aqueous layer was then extracted 3x with ethyl acetate. The organic layers were combined and dried with $MgSO_4$ to yield 120 mg of (76%) of a yellow solid. ^1H NMR (500 MHz, DMSO-d_6) δ 8.03 (t, *J* = 5.7 Hz, 1H), 7.84 (t, *J* = 5.7 Hz, 1H), 7.50 (s, 1H), 7.33 (s, 1H), 6.17 (dd, *J* = 17.1, 10.1 Hz, 1H), 6.03 (dd, *J* = 17.1, 2.3 Hz, 1H), 5.54 (dd, *J* = 10.1, 2.3 Hz, 1H), 5.38 (s, 1H), 5.23 (q, *J* = 6.2 Hz, 1H), 4.01 (t, *J* = 6.5 Hz, 2H), 3.88 (s, 3H), 3.10 (q, *J* = 6.6 Hz, 2H), 3.03 (q, *J* = 6.6 Hz, 2H), 2.49 (s, 1H), 2.21 (t, *J* = 7.4 Hz, 2H), 1.92 (p, *J* = 6.96 Hz, 2H), 1.53 (p, *J* = 7.0 Hz, 2H), 1.34 (d, *J* = 6.3 Hz, 3H). ^{13}C NMR (126 MHz, DMSO-d_6) δ 171.83, 164.99, 153.85, 146.72, 139.35, 138.41, 132.26, 125.36, 109.52, 108.79, 68.74, 64.36, 56.51, 36.89, 36.87, 32.08, 29.66, 25.64, 25.17. IR: ν = 3214, 1737, 1653, 1515, 1267, 1212, 1102, 1018, 804. ESI-MS calculated for $C_{19}H_{27}N_3O_7$ $[M+H]^+$ = 410.1927, observed = 410.1984.

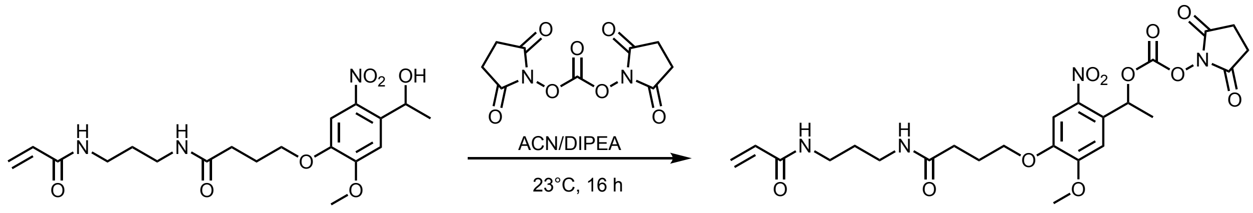

6
(50%)

Synthesis of 6. Alcohol **5** (308 mg, 752 µmol, 1 equivalent) and triethyl amine (524 µL, 3.76 mmol, 5 equivalents) were dissolved in dry acetonitrile in an oven-dried round bottom flask. N,N′-Disuccinimidyl carbonate (964 mg, 3.76 mmol, 5 equivalents) was then added and reaction was allowed to proceed overnight for 16 h. Product was then concentrated, and partially purified via column chromatography with a DCM+4% MeOH mobile phase. Fractions containing the product were combined and concentrated. Residual electrophile was then removed by redissolving in DCM and washing twice with sodium bicarbonate. Product was then dried with $MgSO_4$ and concentrated to yield 205 mg (50%) as a yellow foam. ^1H NMR (600 MHz, chloroform-*d*) δ 7.63 (s, 1H), 7.06 (s, 1H), 6.53 (t, *J* = 6.4 Hz, 1H), 6.48 (q, *J* = 6.4 Hz, 1H), 6.26 (dd, *J* = 17.0, 1.4 Hz, 1H), 6.12 (dd, *J* = 17.0, 10.3 Hz, 1H), 5.64 (dd, *J* = 10.3, 1.4 Hz, 1H), 4.18 – 4.05 (m, 2H), 4.03 (s, 3H), 3.34 –

3.23 (m, 2H), 2.80 (s, 4H), 2.44 (t, $J = 7.2$ Hz, 2H), 2.20 (p, J=6.55, 2H), 1.76 (d, $J = 6.4$ Hz, 3H), 1.63 (p, $J = 6.15$, 2H). ^{13}C NMR (126 MHz, chloroform-d) δ 172.69, 168.40, 166.05, 154.42, 147.59, 139.19, 131.03, 130.82, 126.25, 109.07, 107.23, 76.45, 68.44, 56.41, 35.76, 35.64, 32.67, 29.64, 25.31, 24.70, 21.81. IR: $\nu = 1737, 1365, 1217$.

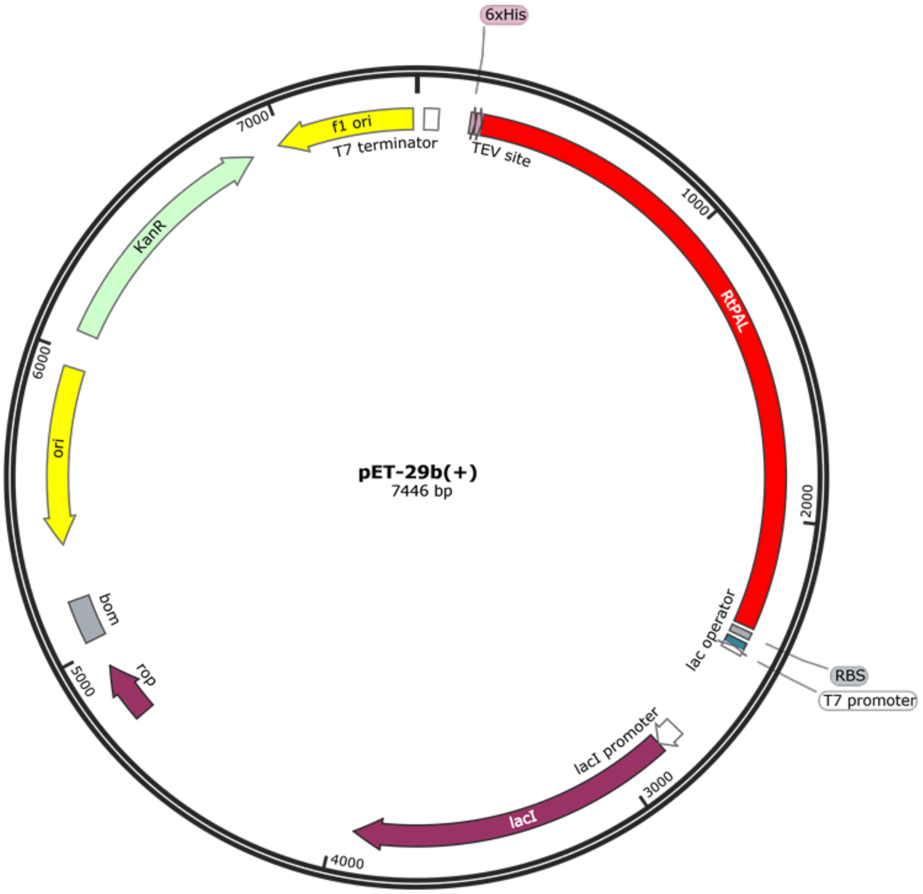

Figure S1. pET-29b Plasmid map for the expression of Phenylalanine Ammonia Lyase

GGATCTGATAAAATTCATCATCATCATCATCACGAAAACCTGTACTTCCAGGGCATG
GCGCCTAGCTTAGACAGCATCTCCCATAGCTTCGCTAATGGCGTTGCATCTGCTAAG
CAAGCAGTTAATGGGGCATCTACTAACTTAGCCGTTGCCGGCTCCCATTTACCTACG
ACGCAAGTAACGCAGGTTGACATCGTGGAAAAGATGCTTGCTGCGCCGACTGACAG
TACCCTTGAGTTAGACGGTTACTCTCTGAATCTTGGGGACGTTGTTAGTGCTGCACG
CAAAGGTCGCCCCGTCCGCGTCAAGGACAGTGATGAAATTCGCTCCAAAATTGATA
AATCAGTGGAATTCTTACGTTCGCAGTTGAGCATGTCGGTTTACGGAGTCACCACAG
GGTTTGGCGGGTCAGCCGATACCCGTACTGAAGATGCGATTTCGTTACAGAAAGCTC
TTCTTGAGCATCAACTTTGTGGCGTTTTGCCTAGTTCTTTCGATTCATTTCGCTTAGG
ACGTGGTCTGGAAAATAGCTTGCCGTTAGAAGTGGTTCGTGGAGCGATGACAATCC
GCGTGAATTCACTTACGCGCGGCCACTCGGCTGTCCGTCTTGTCGTCTTAGAAGCTTT
GACTAATTTTCTTAATCATGGAATTACCCCGATCGTCCCTTTGCGCGGTACAATTAGT
GCTAGCGGAGATTTATCGCCCCTGAGCTACATCGCCGCAGCGATTAGCGGACATCCC
GACTCAAAGGTGCATGTTGTCCATGAAGGTAAGGAAAAGATCCTTTACGCACGCGA
AGCAATGGCTCTGTTTAACCTGGAGCCCGTCGTGTTAGGTCCGAAGGAGGGCCTTGG
CTTAGTAAATGGCACAGCTGTGTCAGCGTCAATGGCGACTCTGGCGTTGCATGATGC
GCACATGCTTTCTCTTTTGTCTCAAAGTCTGACAGCCATGACCGTTGAGGCCATGGTT
GGGCATGCCGGCTCCTTTCACCCATTCTTACATGACGTCACGCGTCCTCATCCAACC
CAGATTGAGGTGGCAGGCAACATCCGCAAGCTGTTAGAGGGGTCCCGCTTCGCGGT
CCATCATGAGGAAGAAGTTAAAGTTAAAGACGACGAGGGCATTCTGCGTCAGGACC
GCTACCCCTTGCGCACAAGTCCTCAGTGGCTTGGACCTCTTGTTTCCGATCTTATTCA
TGCACACGCCGTCCTGACAATTGAAGCGGGTCAAAGCACAACCGACAATCCATTAA
TCGATGTCGAGAACAAGACTTCTCACCACGGTGGCAATTTTCAGGCTGCGGCAGTGG
CGAATACGATGGAAAAAACTCGTCTTGGATTAGCGCAAATTGGGAAGCTGAATTTC
ACCCAGCTGACGGAAATGCTGAACGCAGGTATGAACCGTGGCTTACCATCCTGTTTG
GCCGCAGAGGATCCAAGCCTGTCTTACCACTGTAAAGGTCTGGATATTGCAGCGGCC
GCGTATACCTCGGAATTAGGTCACTTGGCTAACCCAGTGACGACTCATGTGCAGCCT
GCCGAAATGGCAAACCAGGCTGTAAACTCTTTAGCTTTGATCAGTGCCCGTCGCACG
ACTGAGTCAAACGACGTCTTATCGTTGCTGTTGGCGACCCACTTATACTGCGTATTA
CAAGCGATTGATCTGCGTGCGATTGAGTTTGAATTCAAAAAGCAATTCGGACCGGCT
ATCGTAAGCCTTATTGACCAACACTTTGGGTCGGCTATGACTGGCTCTAACTTGCGC
GATGAATTAGTCGAGAAAGTTAACAAGACGTTAGCGAAGCGTCTGGAACAGACGAA
CTCTTACGATCTGGTACCTCGTTGGCACGATGCTTTCTCATTTGCCGCAGGAACCGTT
GTCGAGGTGTTAAGCTCGACATCTTTGTCATTGGCAGCTGTTAACGCATGGAAGGTG
GCAGCGGCGGAGTCAGCTATCAGTCTTACACGCCAAGTTCGTGAGACATTTTGGTCT
GCCGCTTCCACAAGCTCCCCGGCACTGAGTTATTTGAGTCCACGTACTCAGATCCTT
TACGCTTTTGTACGTGAGGAATTGGGCGTAAAAGCACGTCGCGGGGATGTGTTCTTA
GGCAAGCAGGAAGTCACCATCGGCTCTAACGTATCGAAGATTTACGAAGCGATTAA
ATCCGGGCGTATTAACAATGTACTTTTGAAGATGCTGGCCTAATGA

Figure S2. cDNA used for Rt-PAL expression. Yellow highlighted region indicates His_6 tag while the green region indicates the TEV cleavage site. Note that the His-cleaved product contains an N-terminal glycine fragment after His-removal.

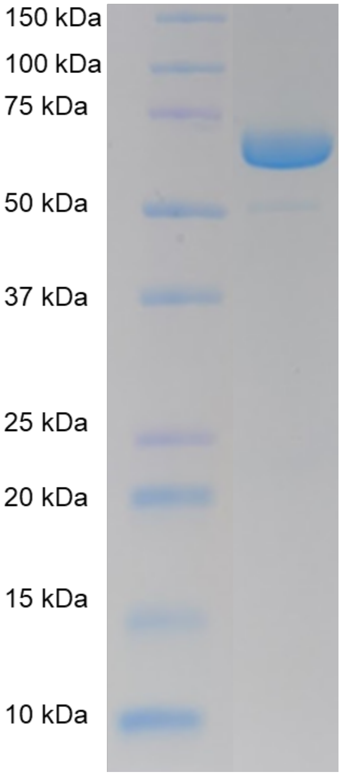

Figure S3. SDS PAGE of His-cleaved PAL product.

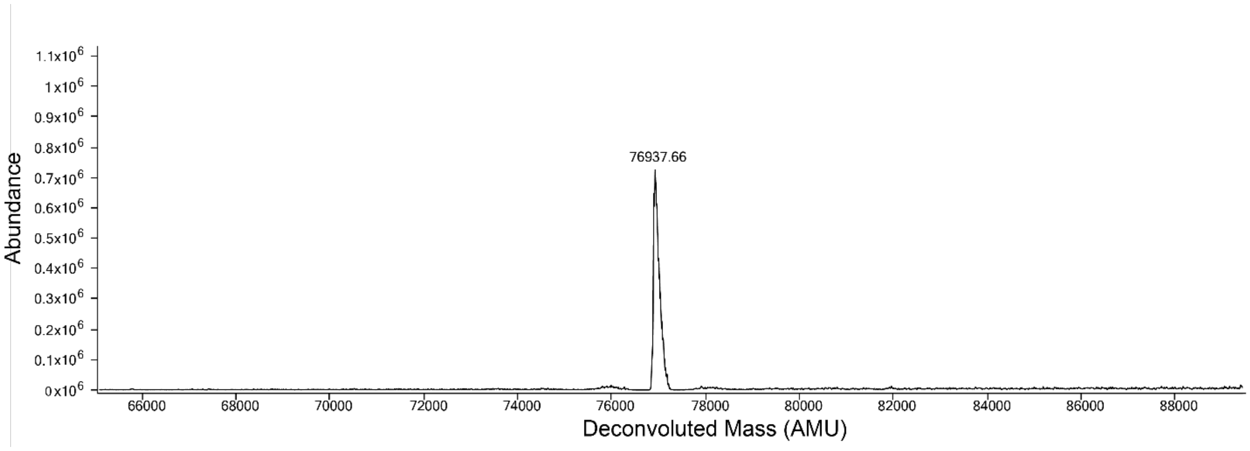

Figure S4. Deconvoluted mass spectrometry of His-cleaved PAL product. Expected mass = 76936.74.

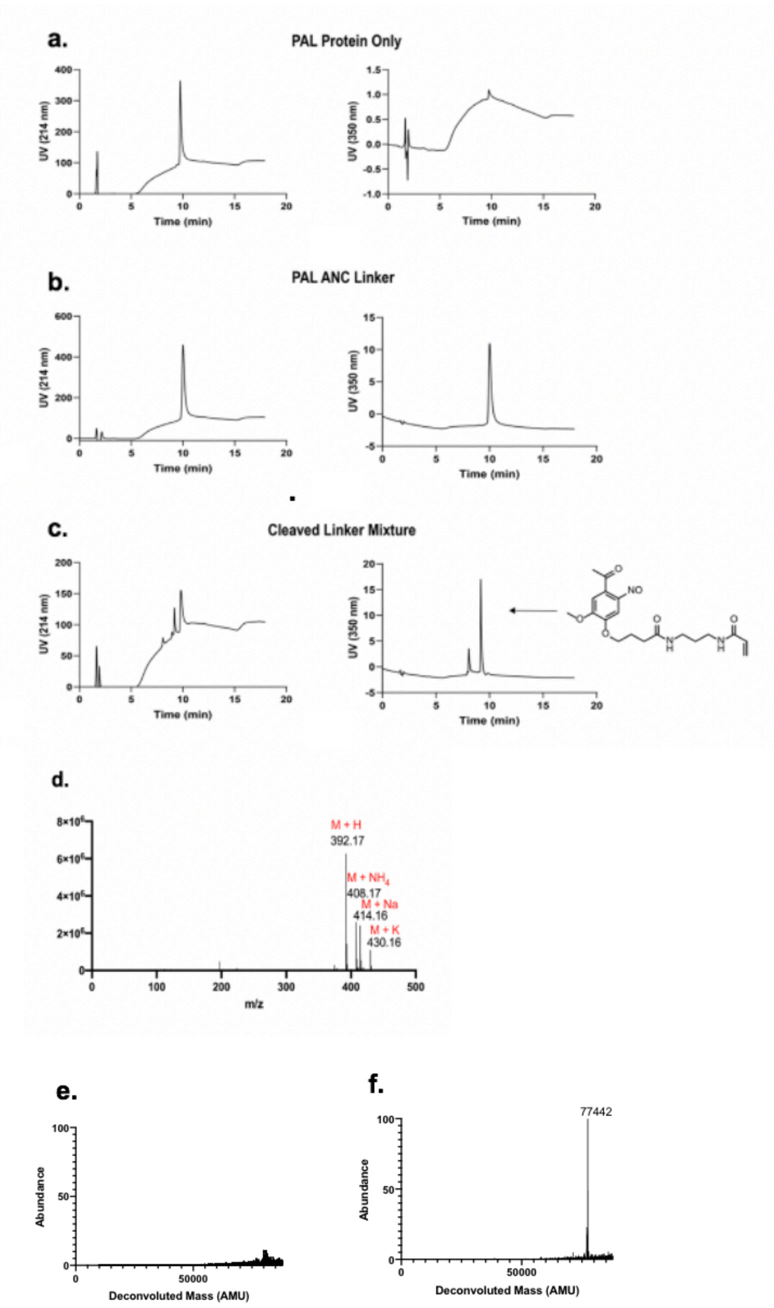

Figures S5. LCMS Chromatograms of (A) PAL at 254 nm (left) and 350 nm (right), (B) ANC-modified PAL at 254 nm (left) and 350 nm (right) UV light, and (C) the crude product of the photocleavage at 254 nm (left) and 350 nm (right). MS Spectrum of (D) photocleavage product (retention time, t = 9.248 min). Expected mass = 392.17. Deconvoluted Protein MS Spectrums of (E) ANC-modified PAL and (F) the crude product of the photocleavage (expected mass of unmodified PAL is 76936).

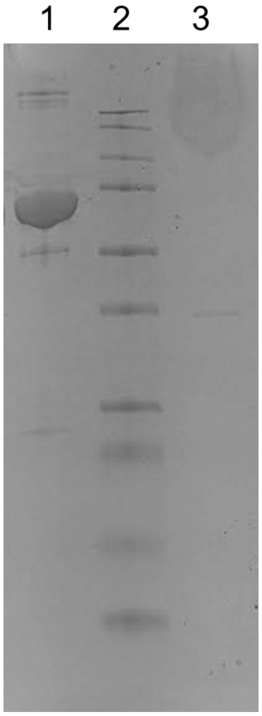

Figure S6. SDS-PAGE of unencapsulated PAL and purified nanogel mixture stained with Coomassie. Lane 1: unencapsulated PAL, lane 2: ladder, lane 3: PAL nanogels (post-irradiation).

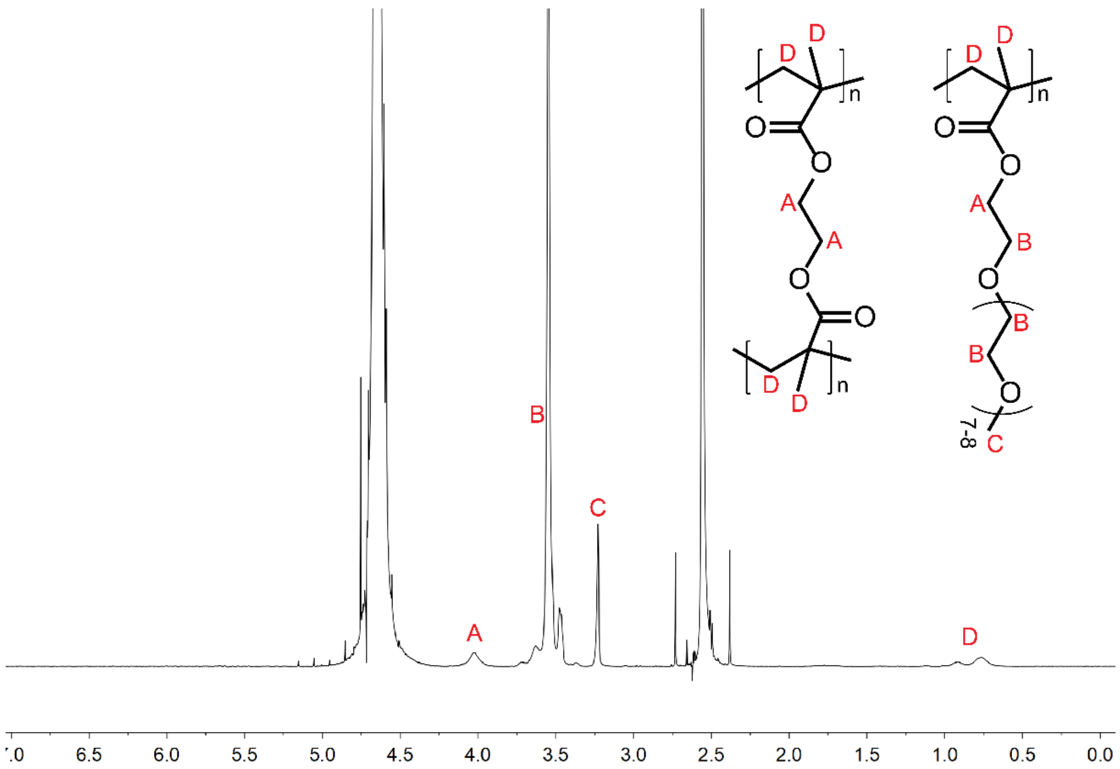

Figure S7. ^{1}H NMR (500 MHz, D2O) of PAL nanogels (post-irradiation).

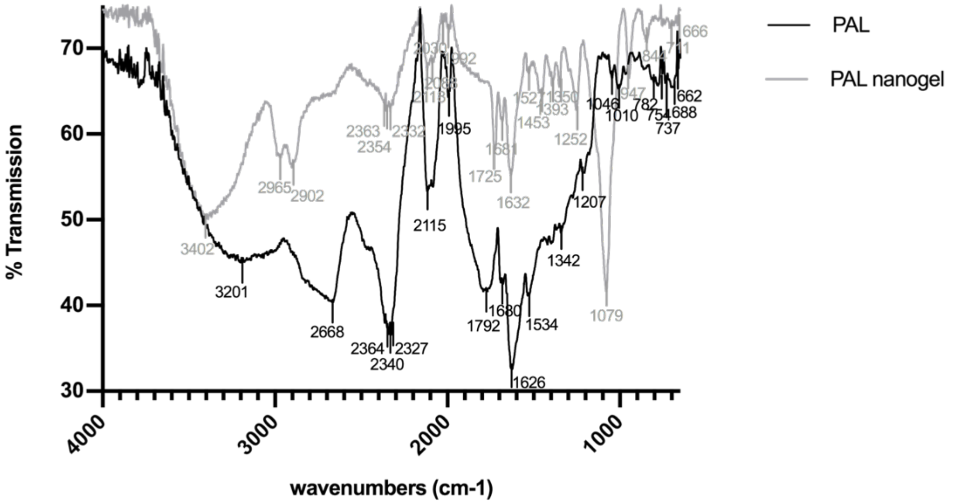

Figure S8. Fourier transform infrared spectrum of PAL and PAL nanogels (post-irradiation).

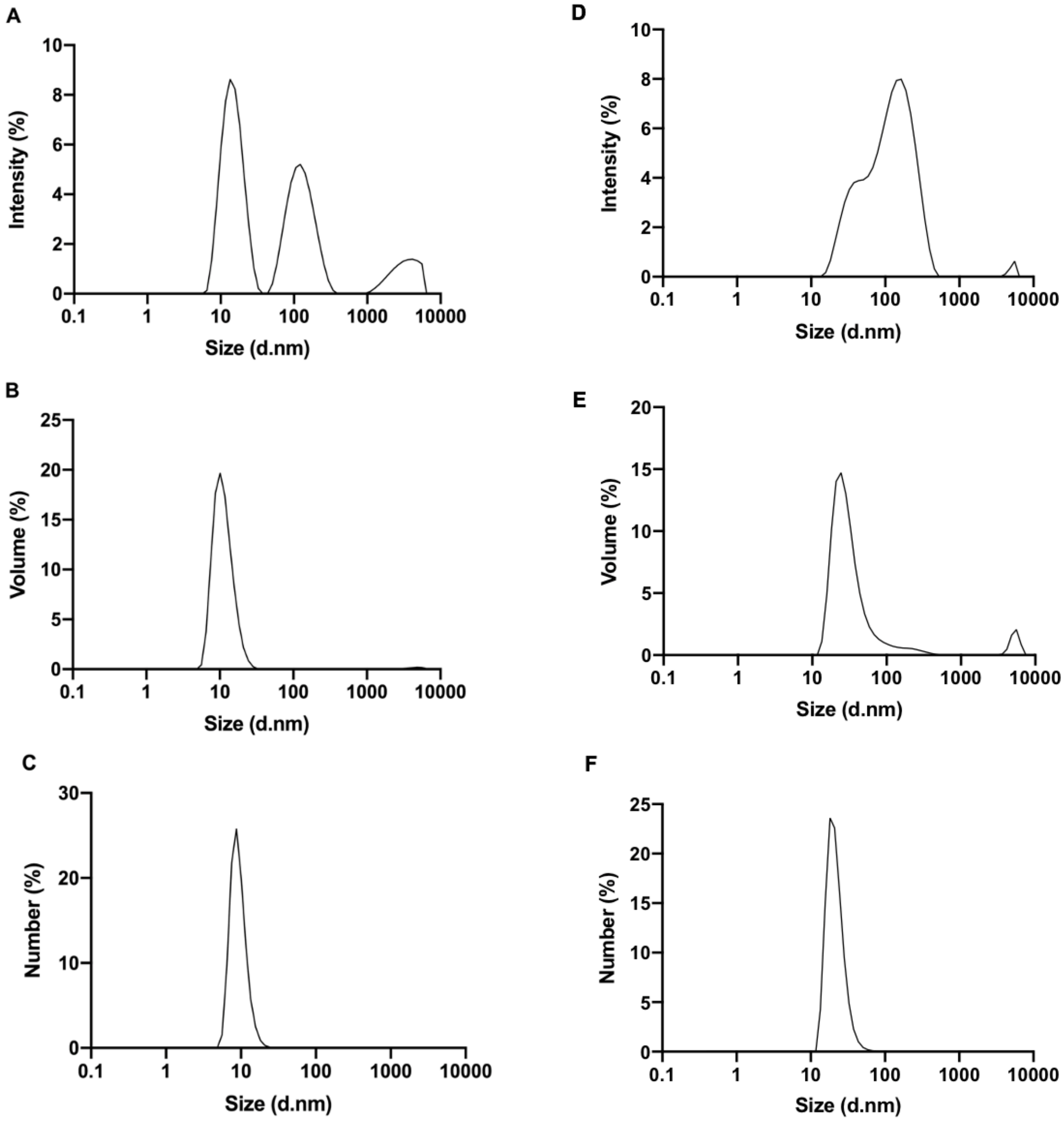

Figure S9. Dynamic Light Scattering of PAL A) Intensity Distribution: Peak 1 – 14.97 d.nm, 50.8%; Peak 2 – 131.8 d.nm, 38.2%; Peak 3 – 3374 d.nm, 10.9% (B) Volume Distribution: Peak 1 – 11.41 d.nm, 98.8%; Peak 2 – 105.0 d.nm, 0.2%; Peak 3 – 4229 d.nm, 0.9% , and (C) Number Distribution: Peak 1 – 9.410 d.nm, 100.0% and of irradiated PAL nanogels. (D) Intensity Distribution: Peak 1 – 142.5 d.nm, 98.9%; Peak 2 – 4968 d.nm, 1.1% (E) Volume Distribution: Peak 1 – 31.39 d.nm, 92.7%; Peak 2 – 273.0 d.nm, 3.1%; Peak 3 – 5161 d.nm, 4.3% (F) Number Distribution: Peak 1 – 20.00 d.nm; 100.0%.

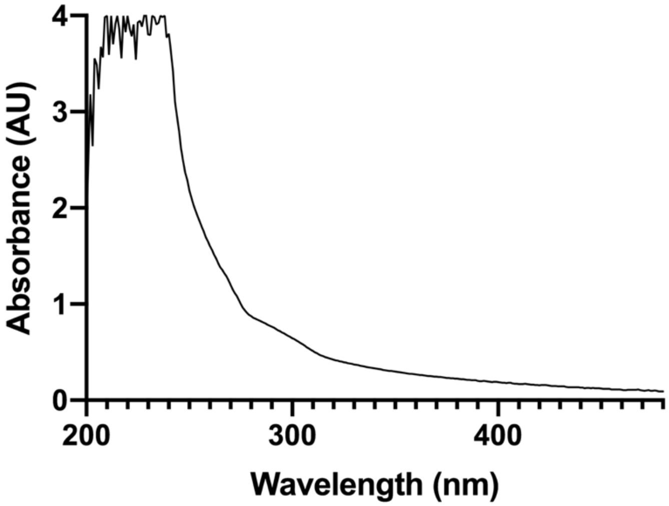

Figure S10. UV-Vis Spectrum of PAL nanogels. Negligible absorbance at 365 nm, which is the wavelength used for photocleavage reaction.

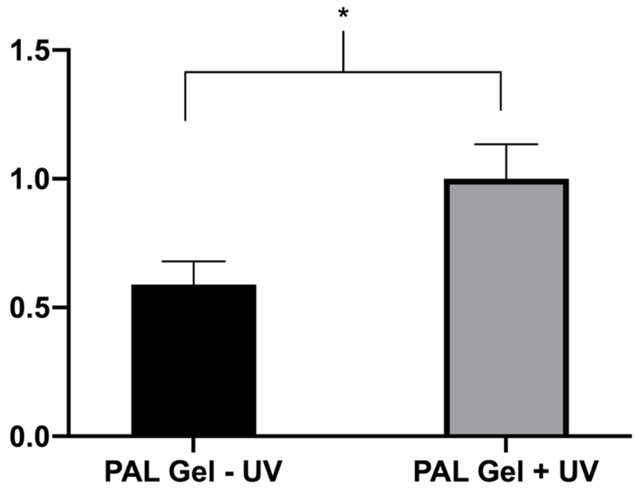

Figure S11. Fluorescamine assay to demonstrate a relative and statistically significant increase in fluorescence after PAL gel is irradiated with UV light.

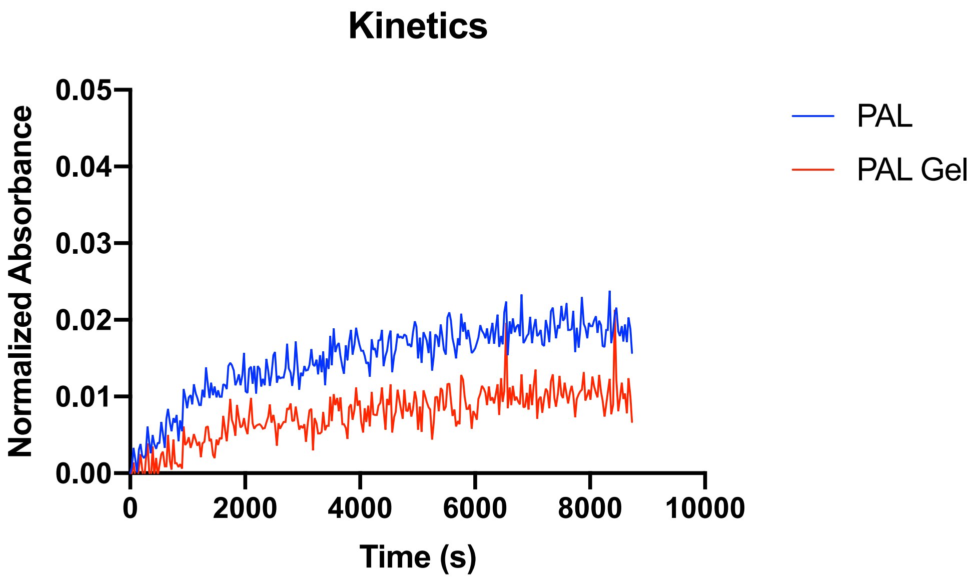

Figure S12. PAL activity kinetics indicate the enzyme reaches its steady state at 2 hours. 2 µg of PAL unencapsulated and freely encapsulated in PAL buffer (50 mM Tris base, 300 mM NaCl pH 7.8 buffer) was added, respectively, to a well in a UV-transparent 96 well plate and diluted up to 180 µL of PAL buffer. Then, 30 µL of 4 mM phenylalanine in PAL buffer was added to initiate the activity assay. Absorbance measurements at 290 nm were taken every 30 seconds and graphed in the figure above.

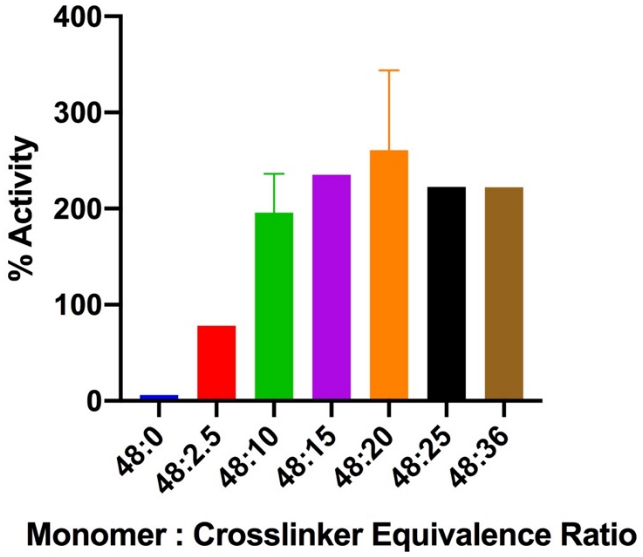

Figure S13. Experiment varying different monomer to crosslinker molar equivalence ratios for PAL nanogels. Eleven individual nanogels were prepared as described previously. All PAL nanogels were exposed to 6 μM trypsin. No significant increase in protein stability was observed for enzymes encapsulated by gels of higher crosslinking densities above a 48:10 monomer to crosslinker ratio.

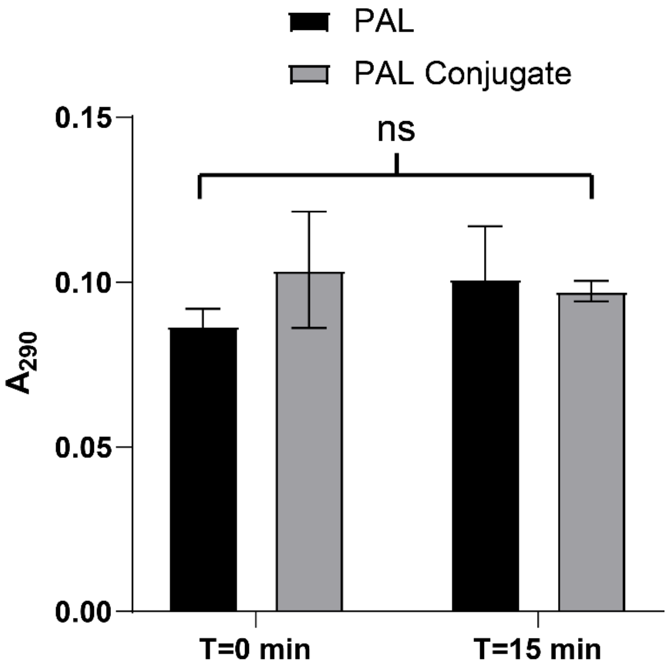

Figure S14. Control experiment verifying that the plate reader assay output is phenylalanine dependent. No statistically significant increase in absorbance at 290 nm was observed over 15 minutes in the absence of phenylalanine.

References

(1) Tropea, J. E.; Cherry, S.; Waugh, D. S. Expression and Purification of Soluble His(6)-Tagged TEV Protease. *Methods Mol. Biol. Clifton NJ* **2009**, *498*, 297–307. https://doi.org/10.1007/978-1-59745-196-3_19.

(2) Kloxin, A. M.; Kasko, A. M.; Salinas, C. N.; Anseth, K. S. Photodegradable Hydrogels for Dynamic Tuning of Physical and Chemical Properties. *Science* **2009**, *324* (5923), 59–63. https://doi.org/10.1126/science.1169494.

^{1}H and ^{13}C NMR

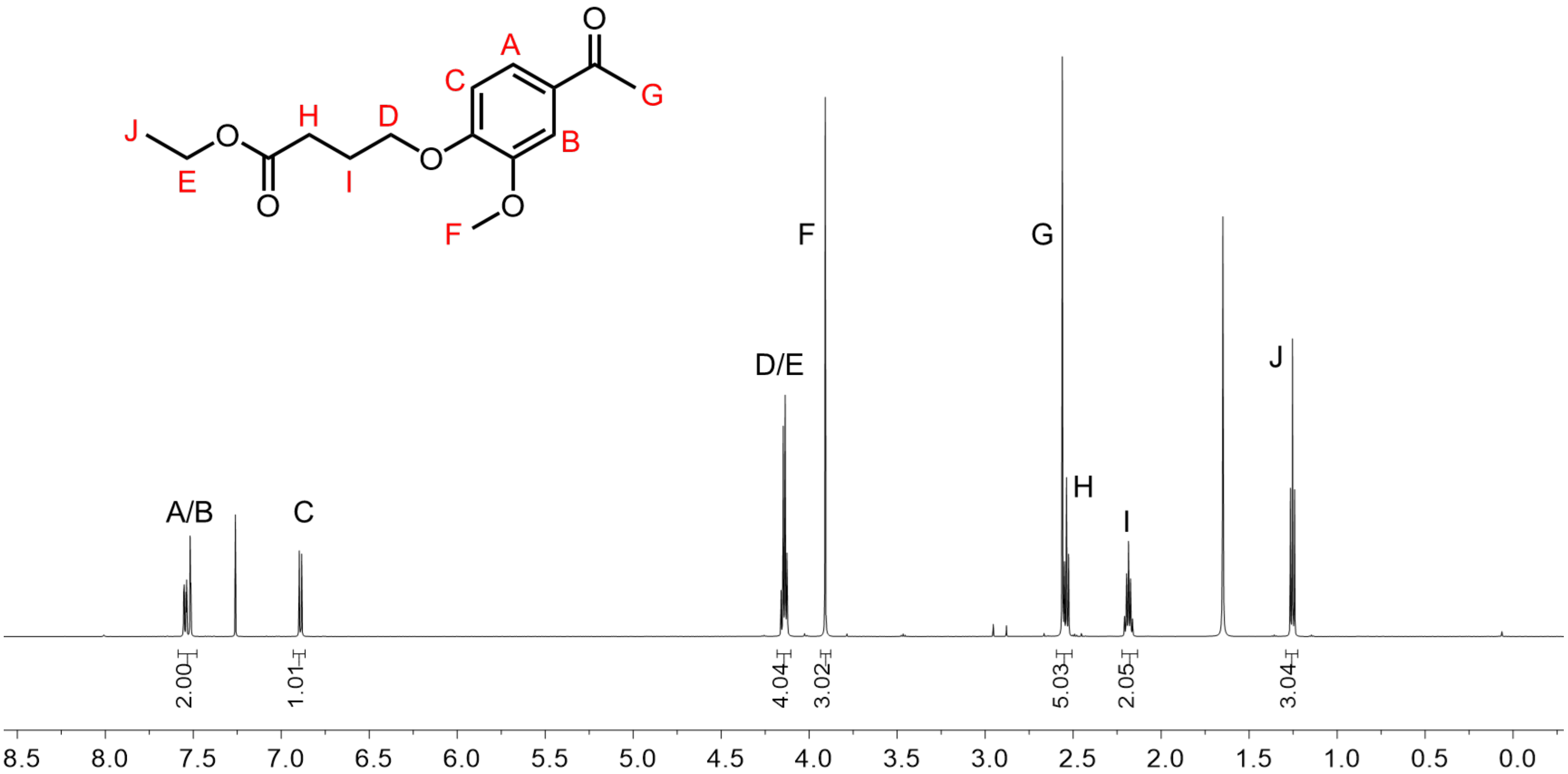

A
C
B
G
H
D
J
E
I
F
A/B
C
D/E
F
G
H
I
J
2.00
1.01
4.04
3.02
5.03
2.05
3.04
8.5 8.0 7.5 7.0 6.5 6.0 5.5 5.0 4.5 4.0 3.5 3.0 2.5 2.0 1.5 1.0 0.5 0.0

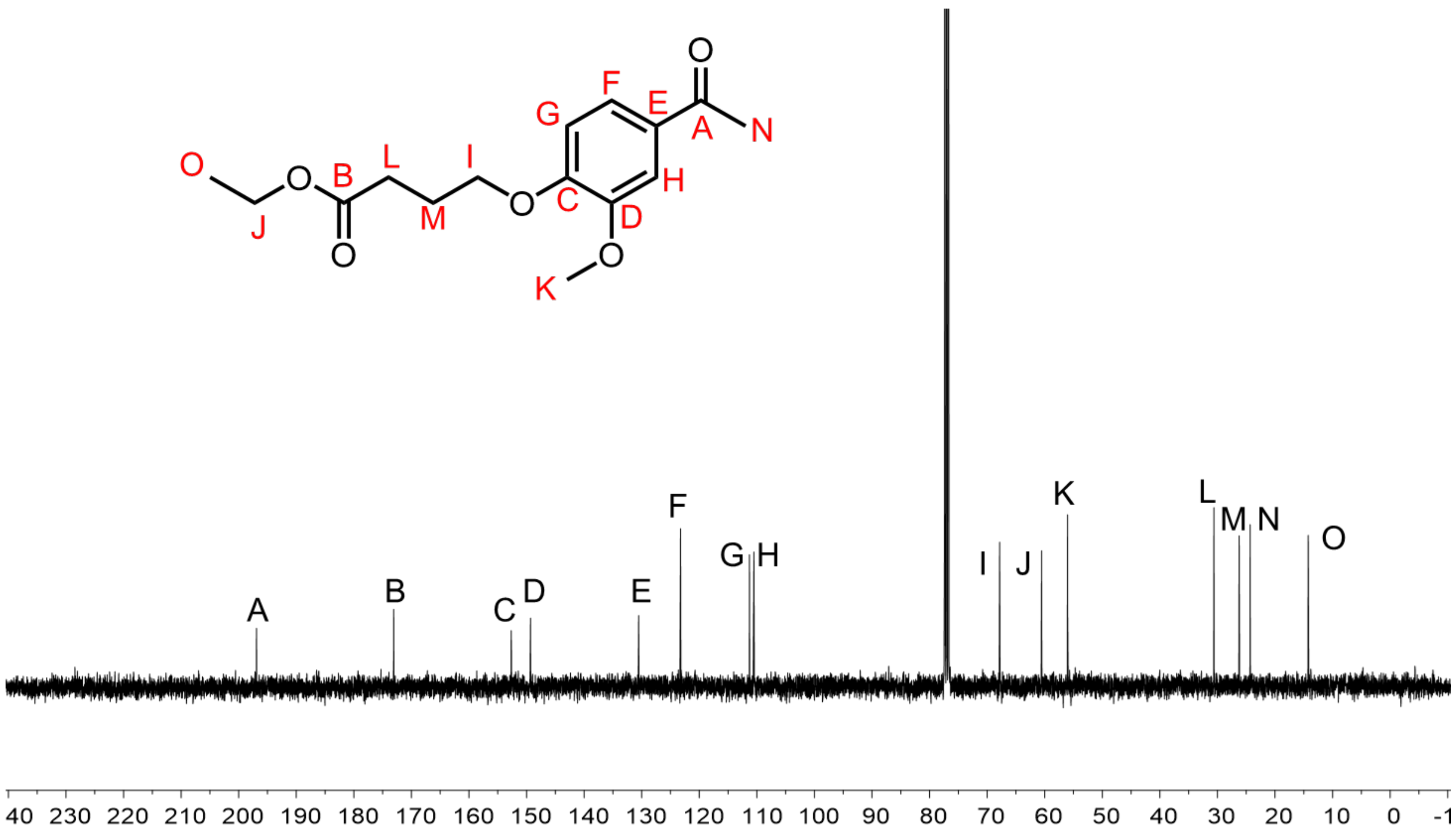

O
B
L
I
F
E
G
A
N
H
C
D
J
M
K
A
B
C
D
E
F
G
H
I
J
K
L
M
N
O
40 230 220 210 200 190 180 170 160 150 140 130 120 110 100 90 80 70 60 50 40 30 20 10 0 -1

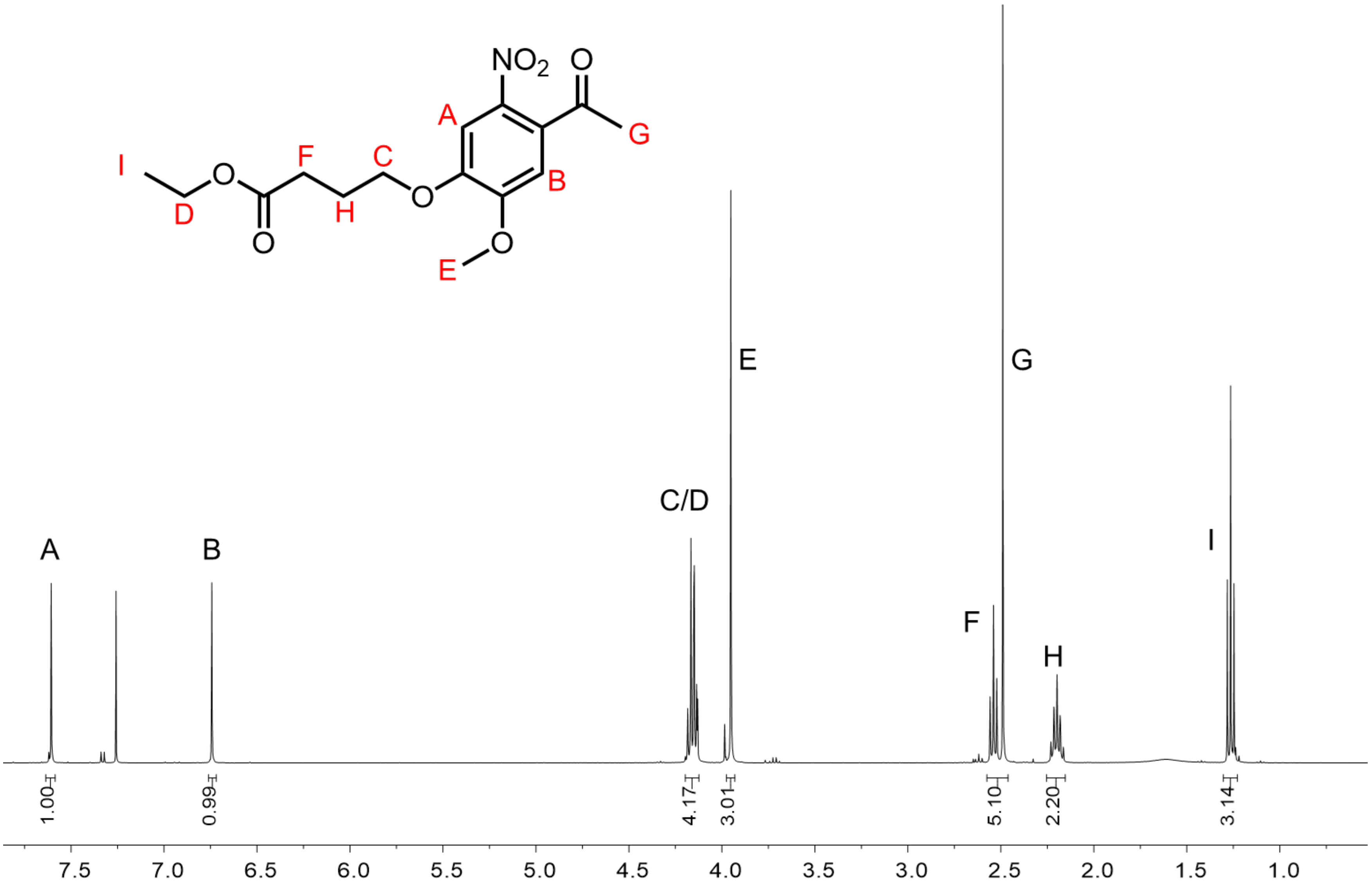
NO2
O
G
A
B
I
F
C
O
O
D
H
O
O
E
A
B
C/D
E
G
F
H
I
1.00
0.99
4.17
3.01
5.10
2.20
3.14
7.5
7.0
6.5
6.0
5.5
5.0
4.5
4.0
3.5
3.0
2.5
2.0
1.5
1.0

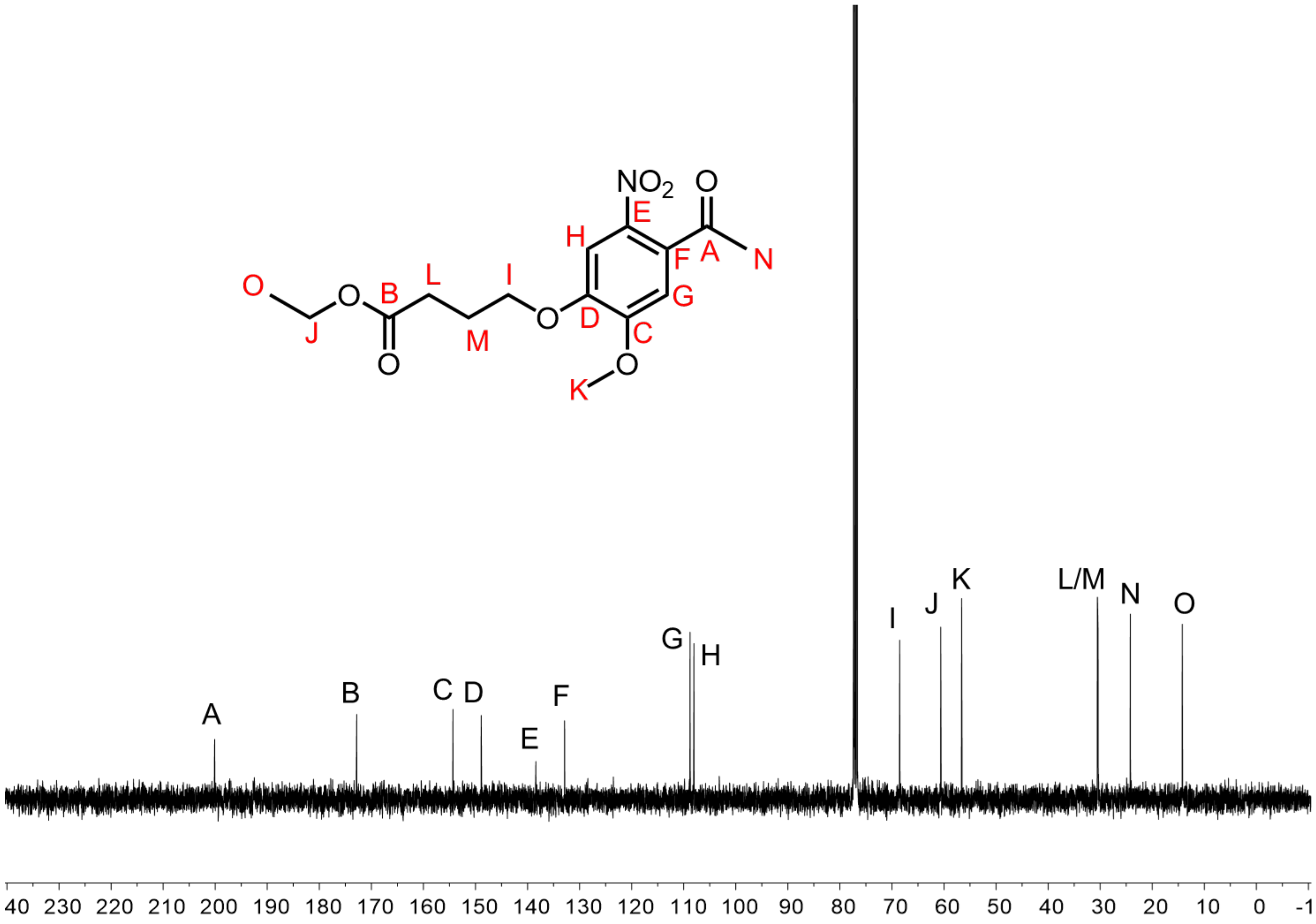

NO2
O
E
H
A
N
F
O
B
L
I
O
G
D
C
J
M
O
O
K
A
B
C
D
E
F
G
H
I
J
K
L/M
N
O
40
230
220
210
200
190
180
170
160
150
140
130
120
110
100
90
80
70
60
50
40
30
20
10
0
-1

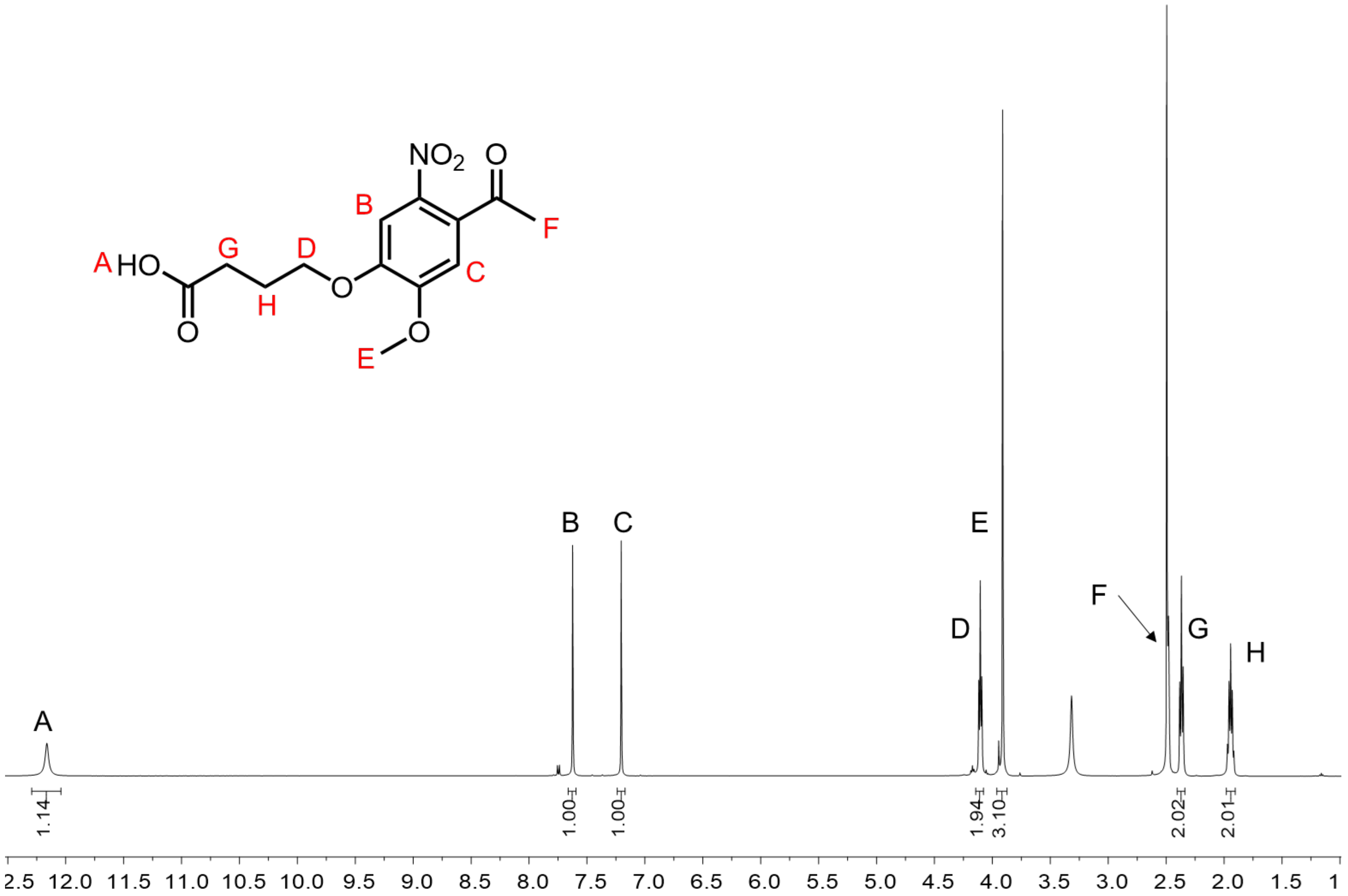

NO2
O
F
B
C
A HO
G
D
H
O
O
O
E
A
B
C
E
D
F
G
H
1.14
1.00
1.00
1.94
3.10
2.02
2.01
2.5 12.0 11.5 11.0 10.5 10.0 9.5 9.0 8.5 8.0 7.5 7.0 6.5 6.0 5.5 5.0 4.5 4.0 3.5 3.0 2.5 2.0 1.5 1

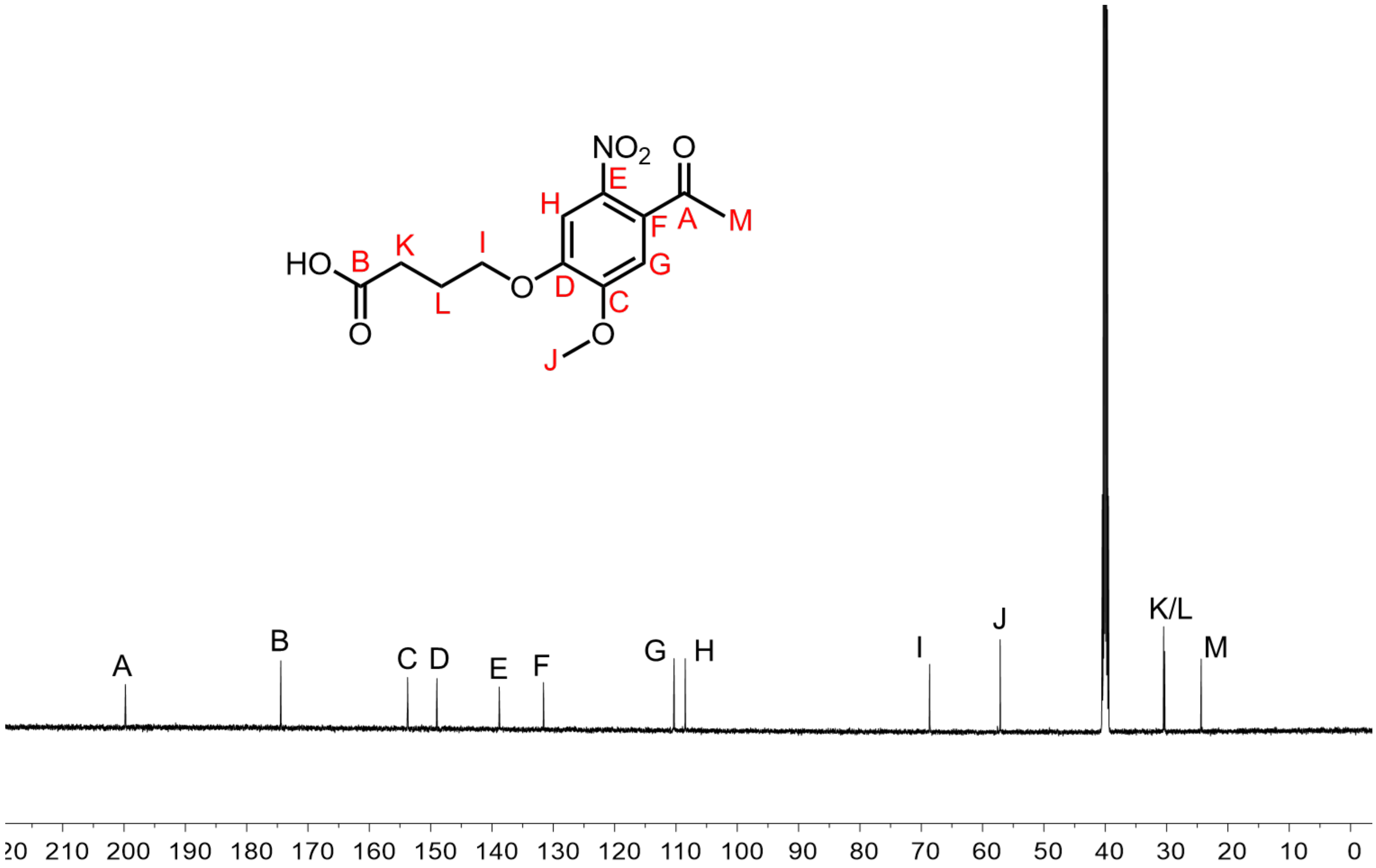
NO2
O
HO
O
O
O
A B C D E F G H I J K L M
K/L
220 210 200 190 180 170 160 150 140 130 120 110 100 90 80 70 60 50 40 30 20 10 0

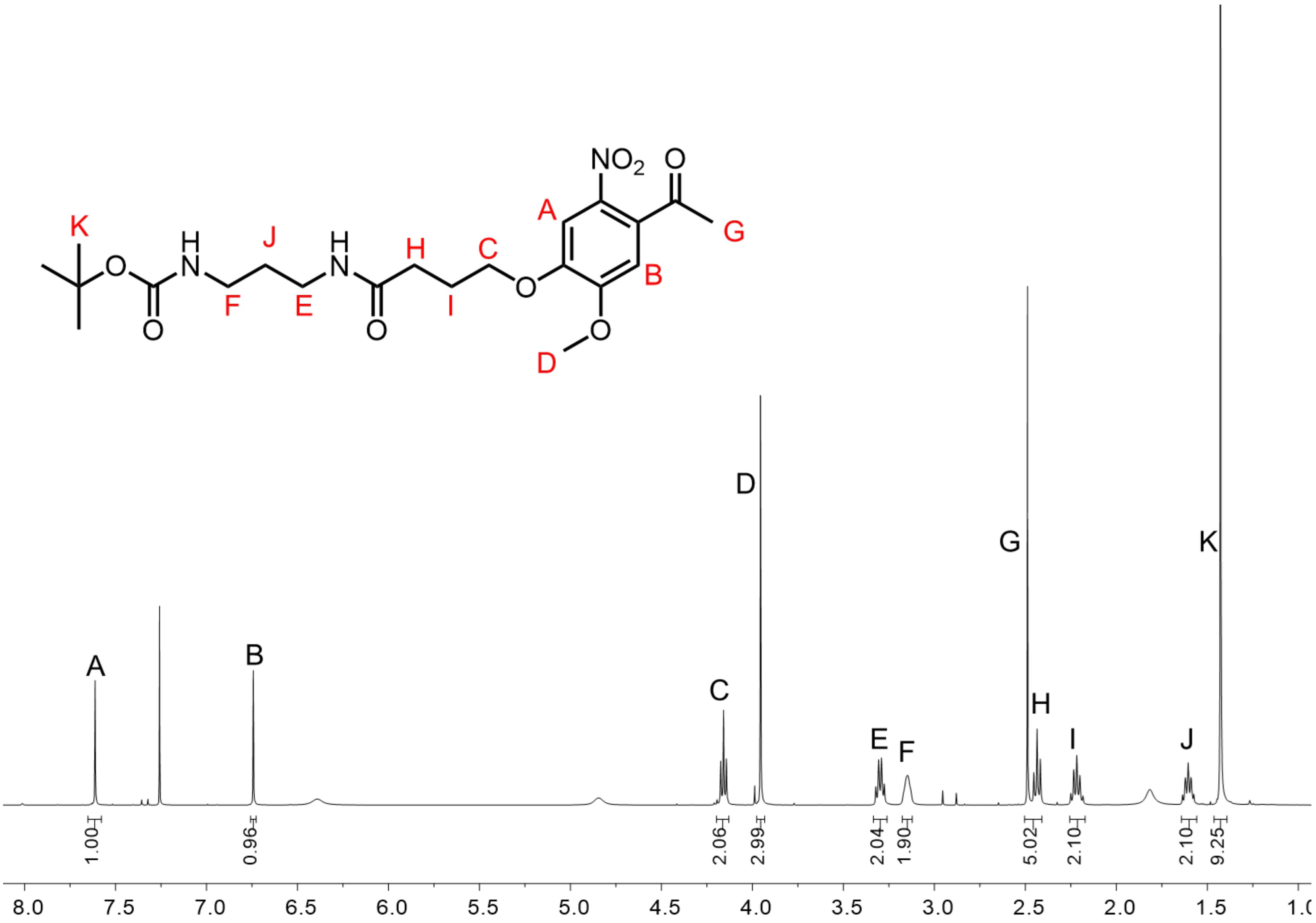

K
H
N
J
H
N
H
C
A
NO2
O
G
B
O
O
F
E
O
I
O
D
A
B
C
D
E
F
G
H
I
J
K
1.00
0.96
2.06
2.99
2.04
1.90
5.02
2.10
2.10
9.25
8.0
7.5
7.0
6.5
6.0
5.5
5.0
4.5
4.0
3.5
3.0
2.5
2.0
1.5

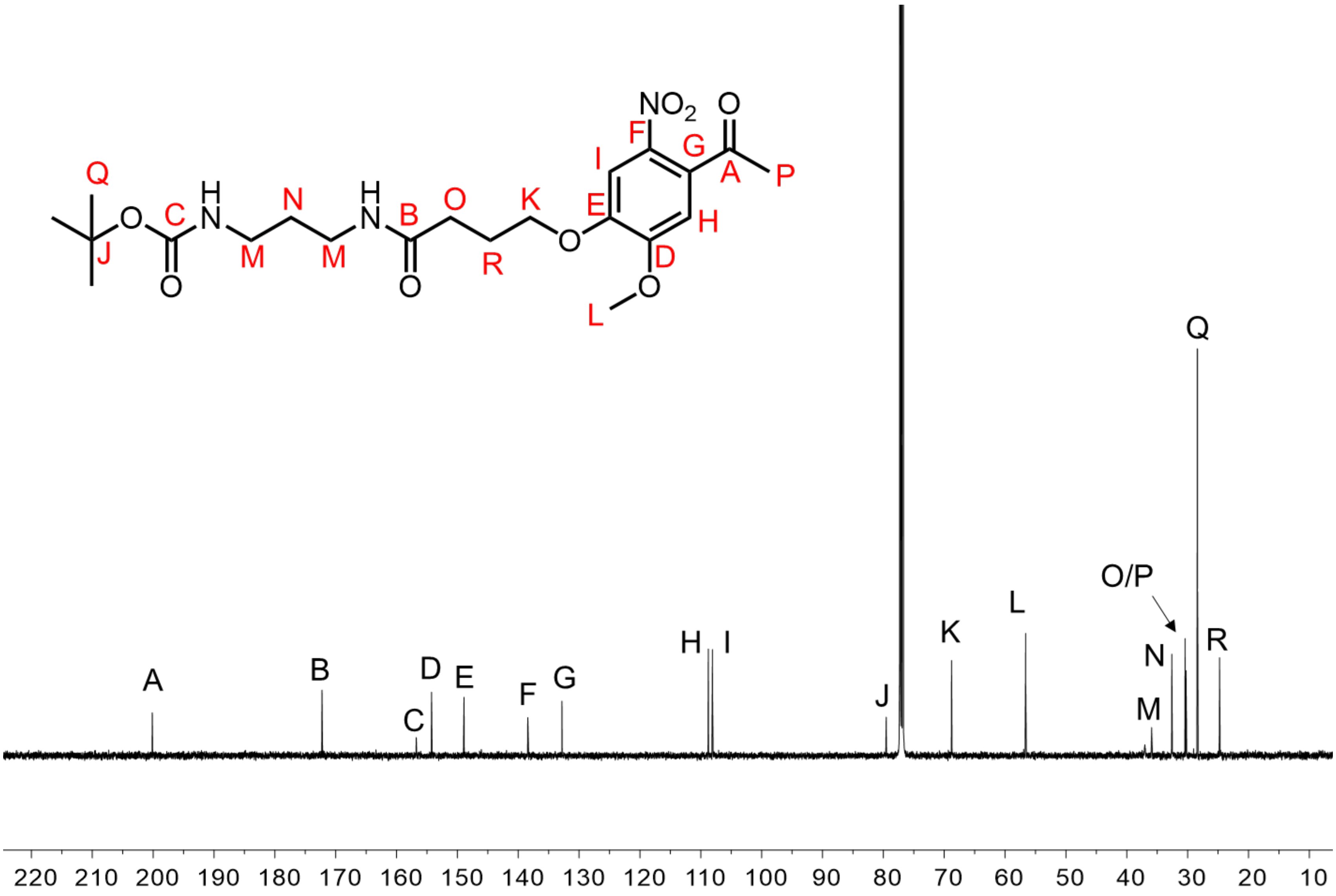

Q
J
C
N
M
B
O
R
K
E
I
F
NO2
G
A
P
H
D
L
A
B
C
D
E
F
G
H
I
J
K
L
O/P
N
M
Q
R
220 210 200 190 180 170 160 150 140 130 120 110 100 90 80 70 60 50 40 30 20 10

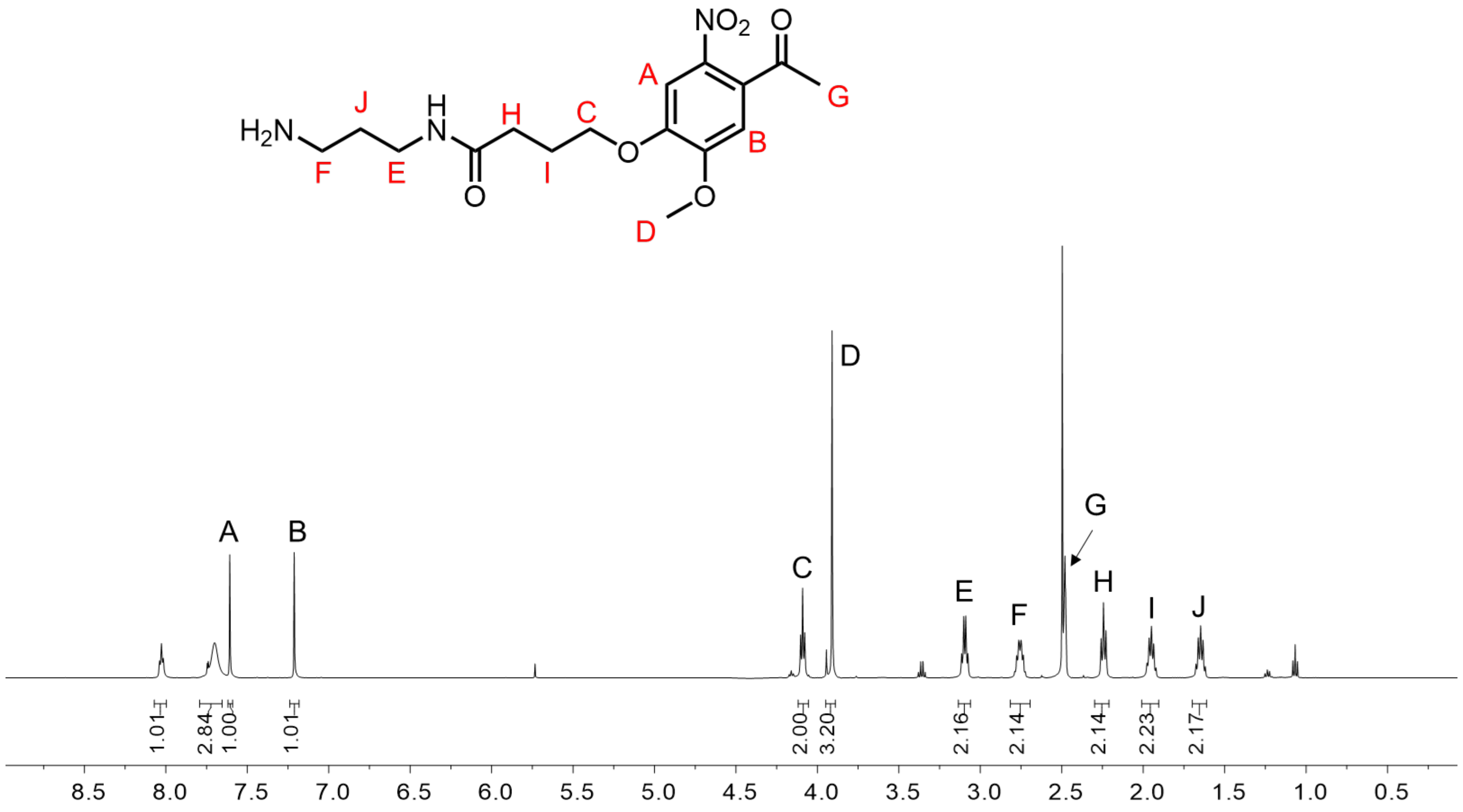

NO2
O
A
G
J
H
H
C
H2N
N
O
B
F
E
I
O
O
D
D
A
B
G
C
E
F
H
I
J
1.01
2.84
1.00
1.01
2.00
3.20
2.16
2.14
2.14
2.23
2.17
8.5
8.0
7.5
7.0
6.5
6.0
5.5
5.0
4.5
4.0
3.5
3.0
2.5
2.0
1.5
1.0
0.5

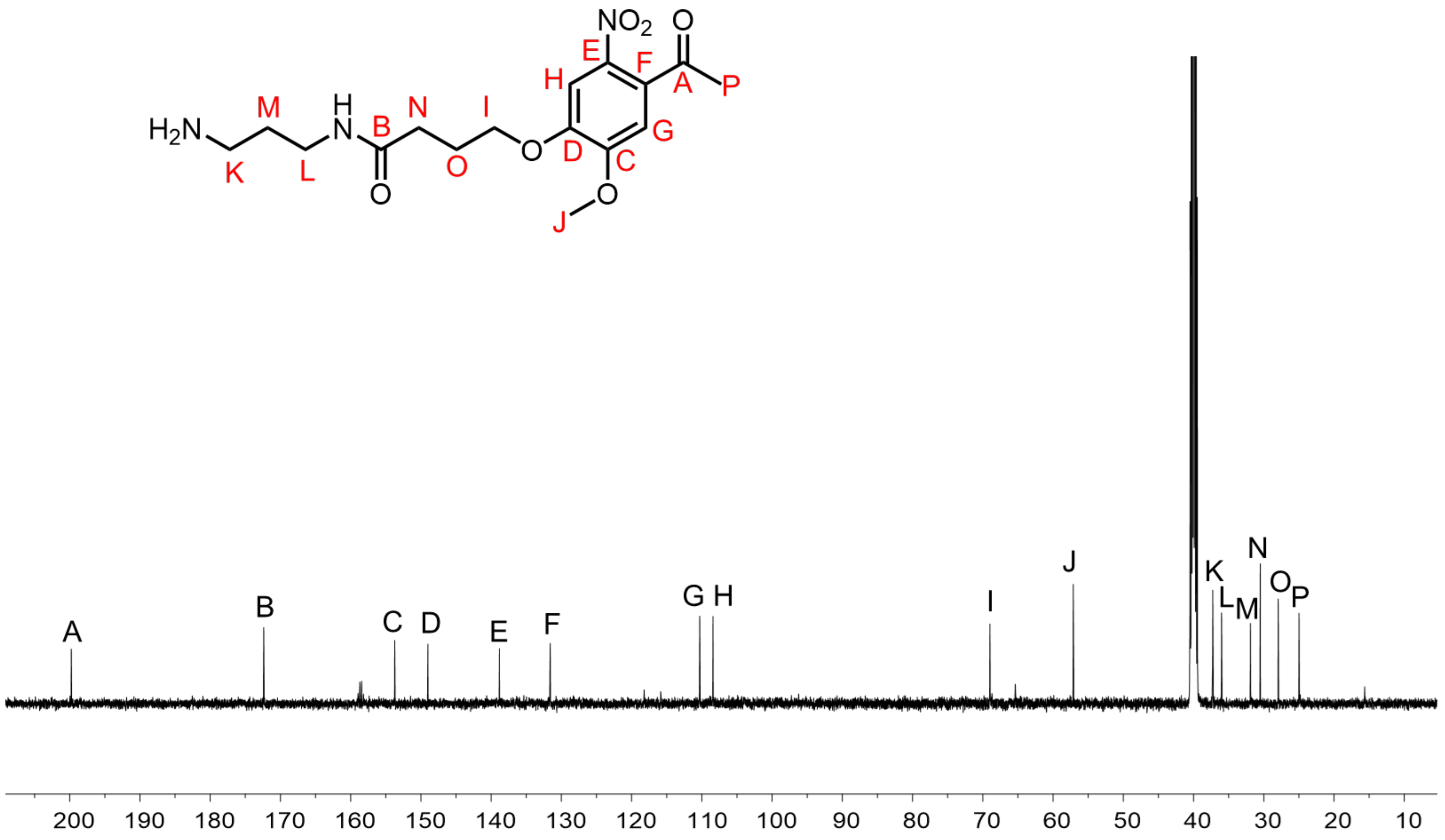
NO2
H2N
O
A
B
C
D
E
F
G
H
I
J
K
L
M
N
O
P
200
190
180
170
160
150
140
130
120
110
100
90
80
70
60
50
40
30
20
10

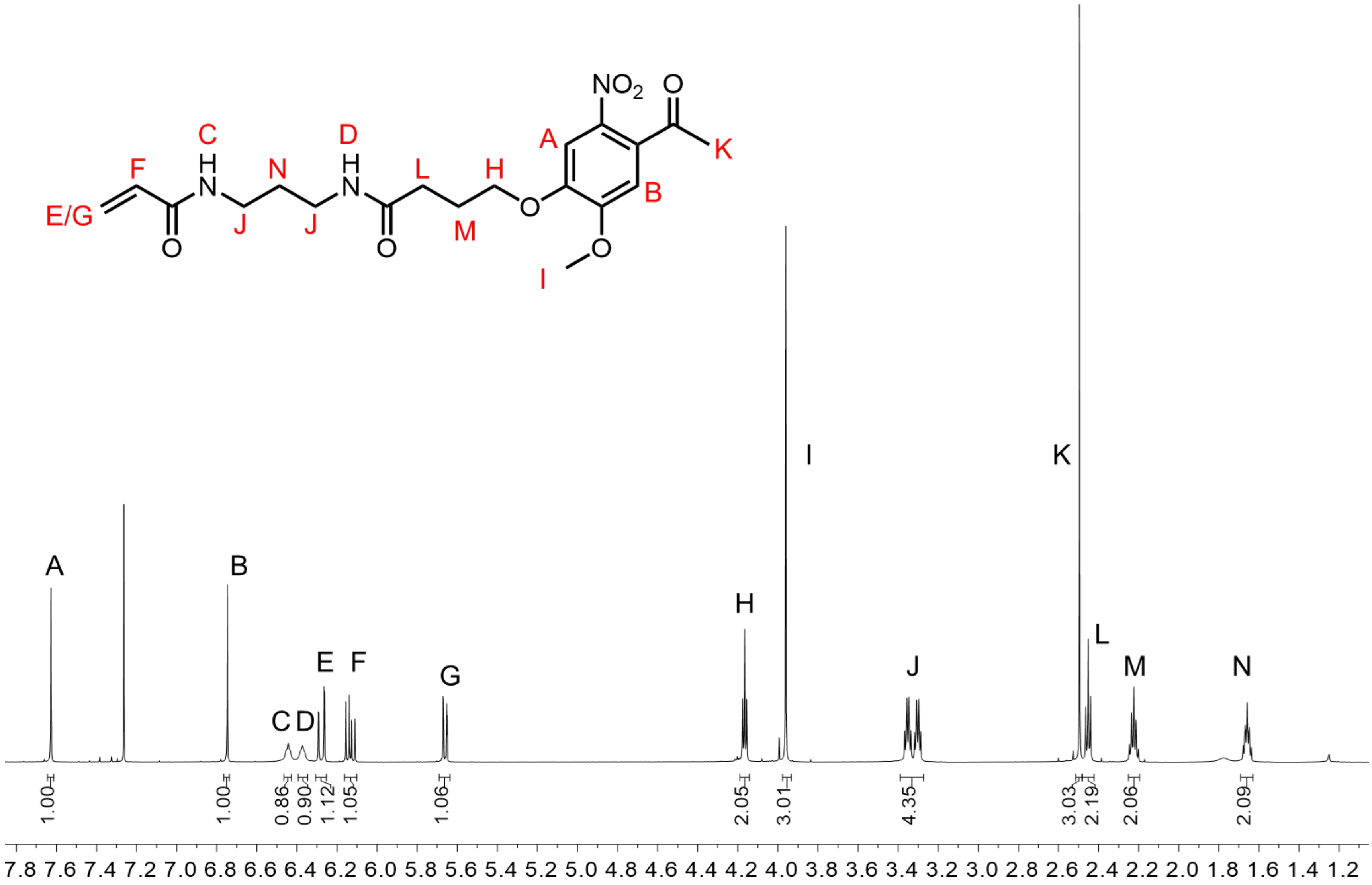

NO2
O
A
K
C
D
F
N
L
H
B
E/G
J
J
M
O
O
I
A
B
C D
E F
G
H
I
J
K
L
M
N
1.00
1.00
0.86
0.90
1.12
1.05
1.06
2.05
3.01
4.35
3.03
2.19
2.06
2.09
7.8 7.6 7.4 7.2 7.0 6.8 6.6 6.4 6.2 6.0 5.8 5.6 5.4 5.2 5.0 4.8 4.6 4.4 4.2 4.0 3.8 3.6 3.4 3.2 3.0 2.8 2.6 2.4 2.2 2.0 1.8 1.6 1.4 1.2

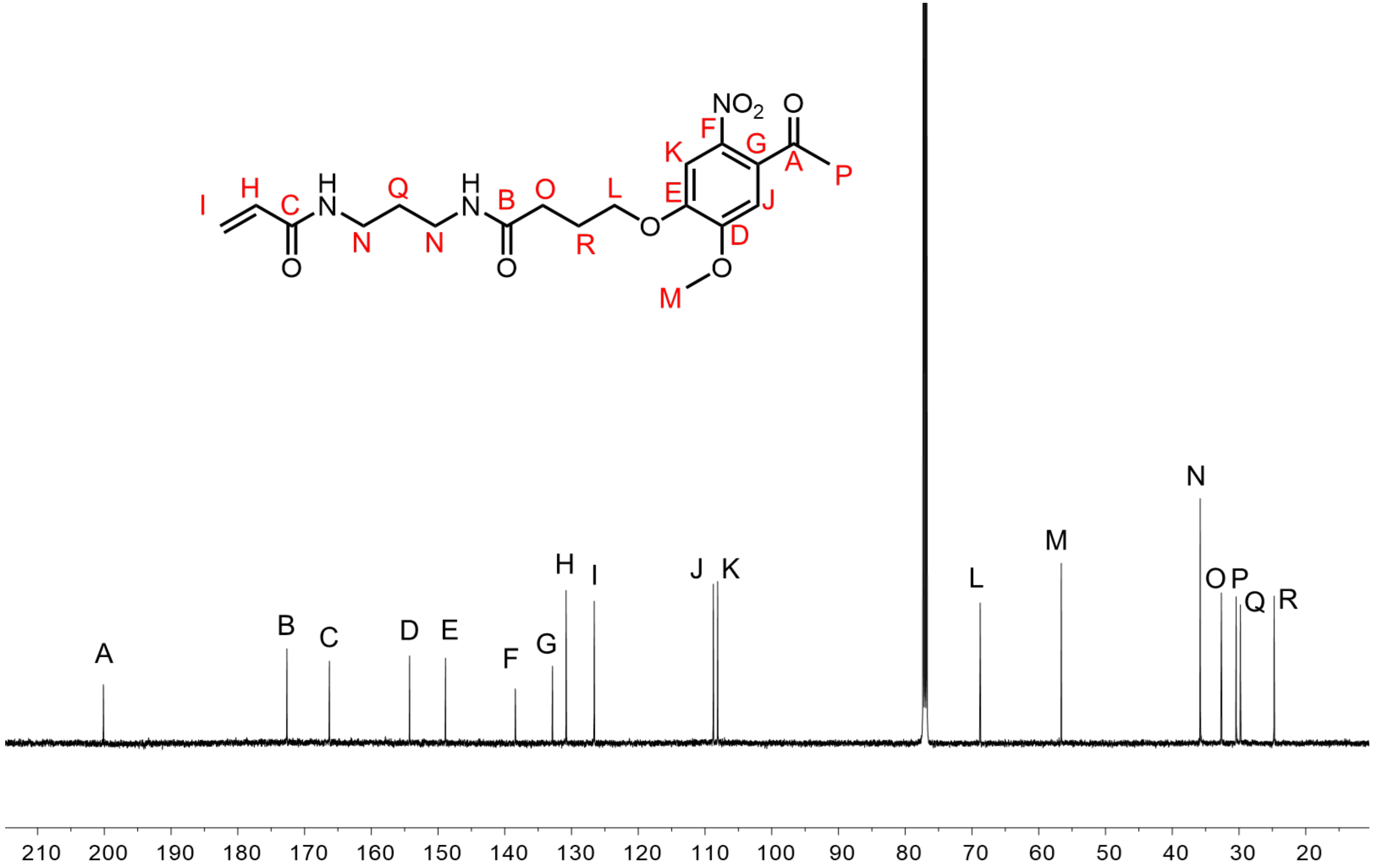
NO2
O
F
G
K
A
P
I
H
C
H
N
Q
H
N
B
O
L
E
J
O
D
N
N
R
O
O
M
O
A
B
C
D
E
F
G
H
I
J
K
L
M
N
O
P
Q
R
210
200
190
180
170
160
150
140
130
120
110
100
90
80
70
60
50
40
30
20

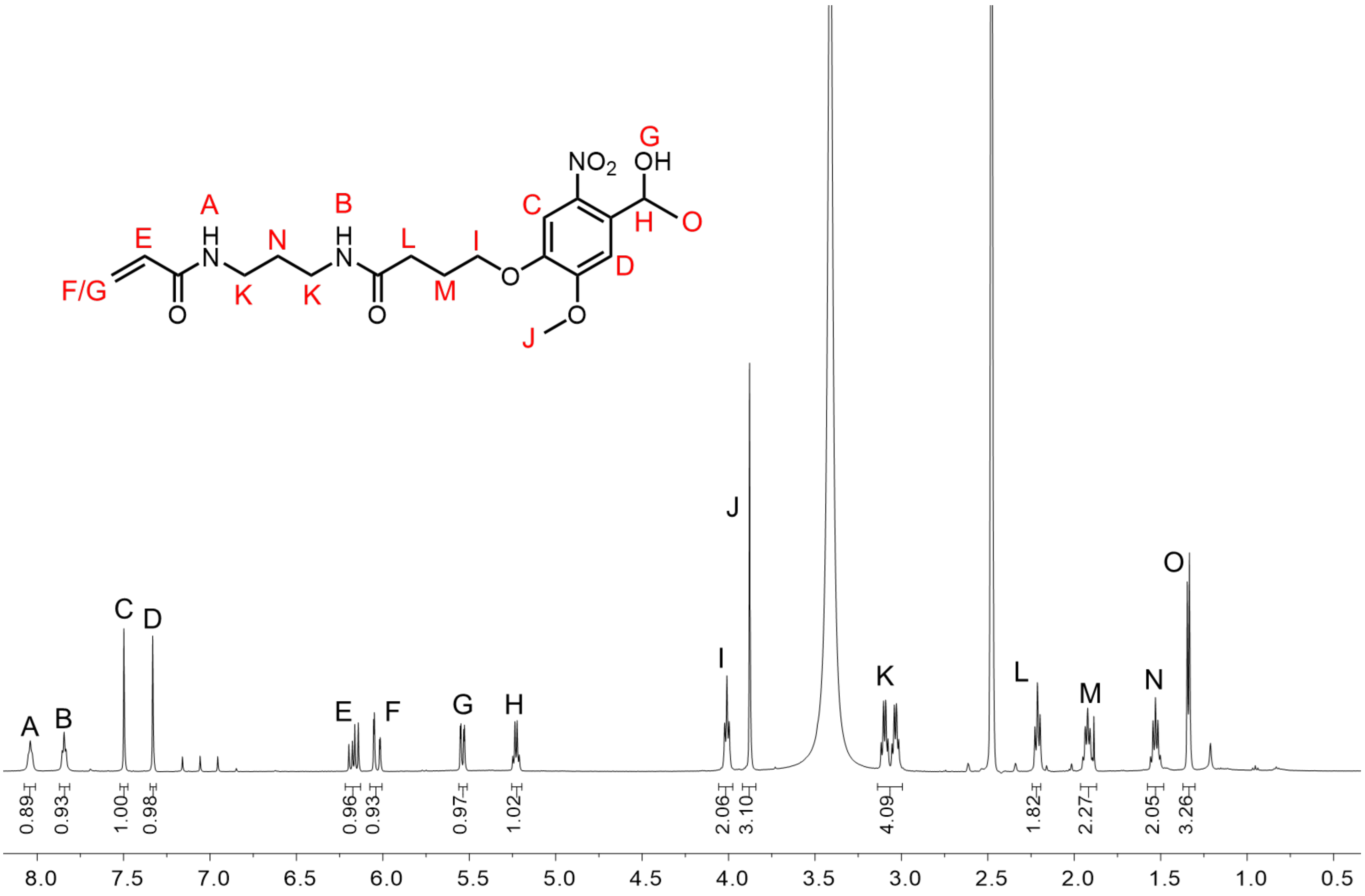

F/G
E
A
N
B
L
I
C
NO_2
G
OH
H
O
D
K
K
M
J
A
B
C
D
E
F
G
H
I
J
K
L
M
N
O
0.89
0.93
1.00
0.98
0.96
0.93
0.97
1.02
2.06
3.10
4.09
1.82
2.27
2.05
3.26
8.0
7.5
7.0
6.5
6.0
5.5
5.0
4.5
4.0
3.5
3.0
2.5
2.0
1.5
1.0
0.5

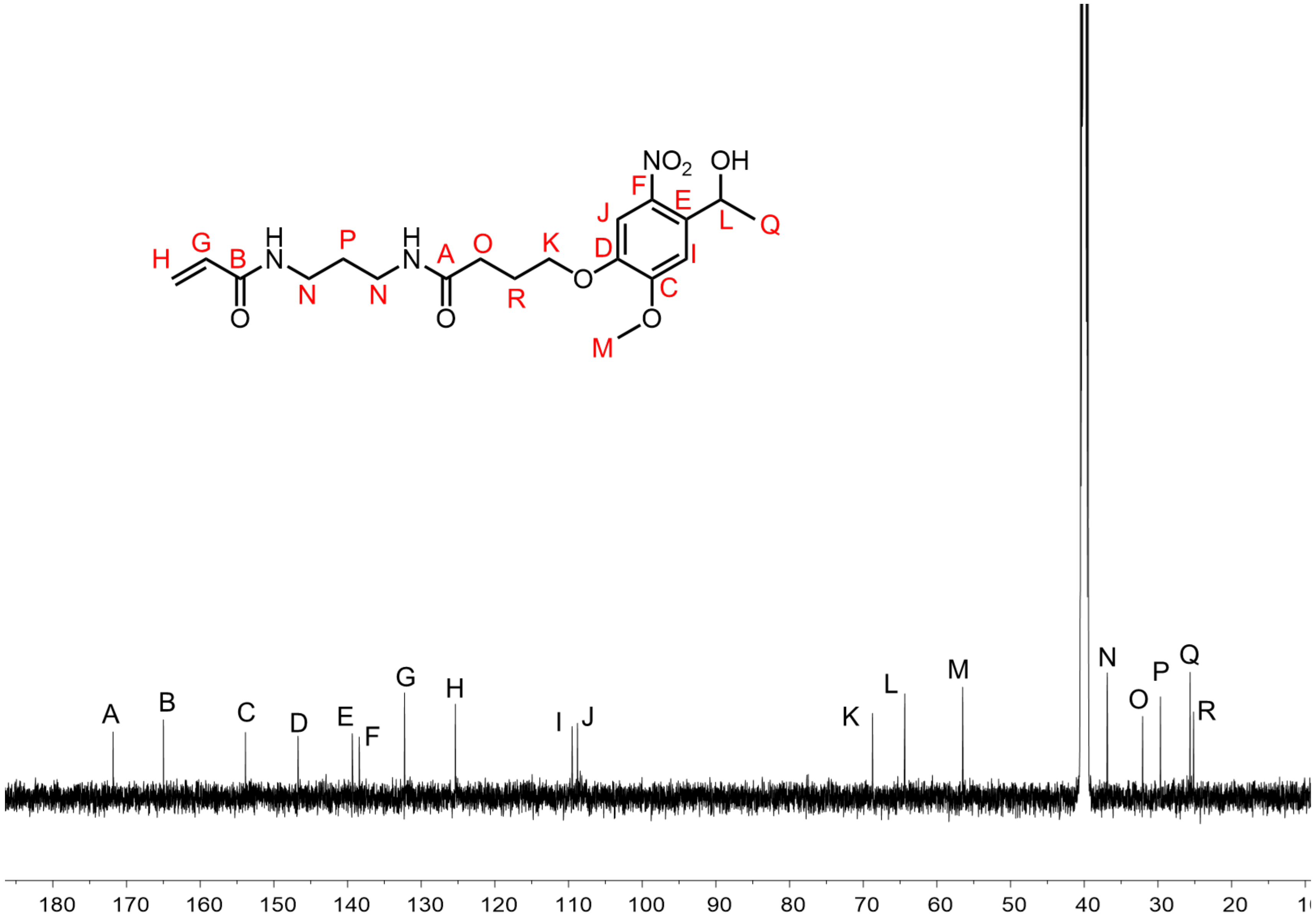
NO2
OH
H
N
H
N
O
O
O
A
B
C
D
E
F
G
H
I
J
K
L
M
N
O
P
Q
R
180
170
160
150
140
130
120
110
100
90
80
70
60
50
40
30
20

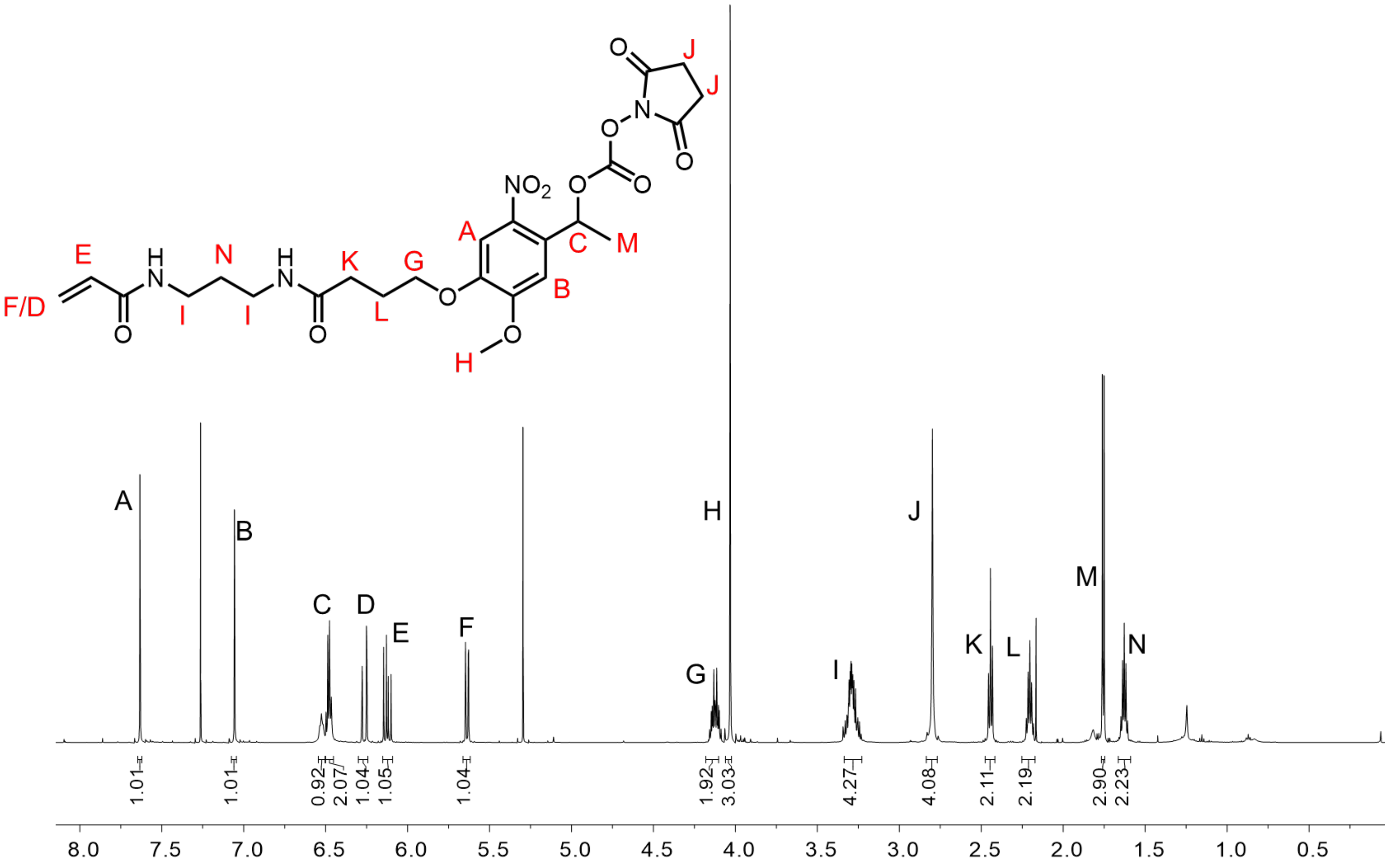
NO2
A
B
C
M
E
F/D
N
K
G
I
L
H
J
A
B
C
D
E
F
G
H
I
J
K
L
M
N
1.01
1.01
0.92
2.07
1.04
1.05
1.04
1.92
3.03
4.27
4.08
2.11
2.19
2.90
2.23
8.0
7.5
7.0
6.5
6.0
5.5
5.0
4.5
4.0
3.5
3.0
2.5
2.0
1.5
1.0
0.5

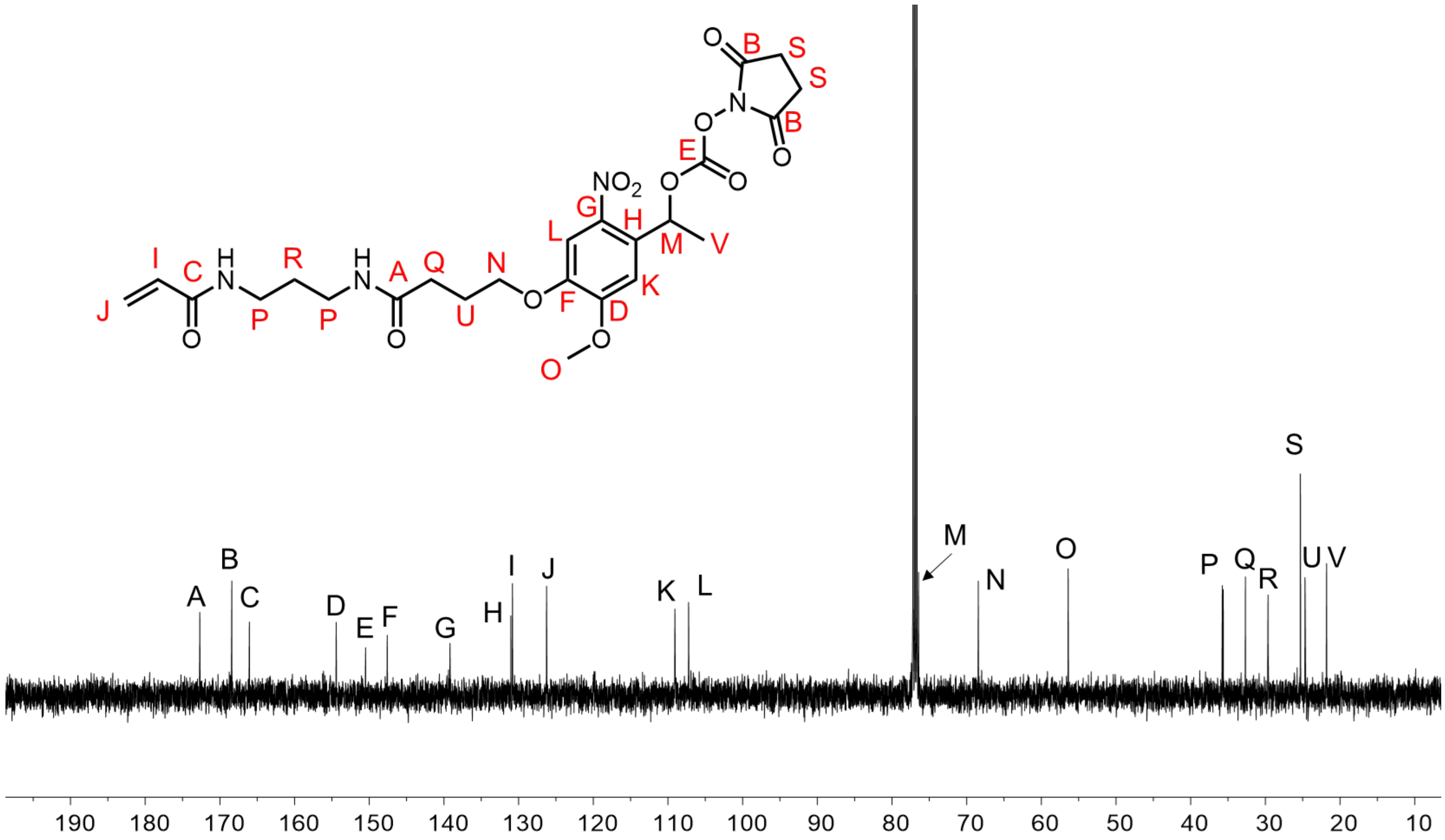

NO2
O
N
H
S
B
E
G
L
I
C
R
A
Q
N
M
V
K
J
P
P
U
F
D
O
S
A
B
C
D
E
F
G
H
I
J
K
L
M
N
O
P
Q
R
U
V
190
180
170
160
150
140
130
120
110
100
90
80
70
60
50
40
30
20
10

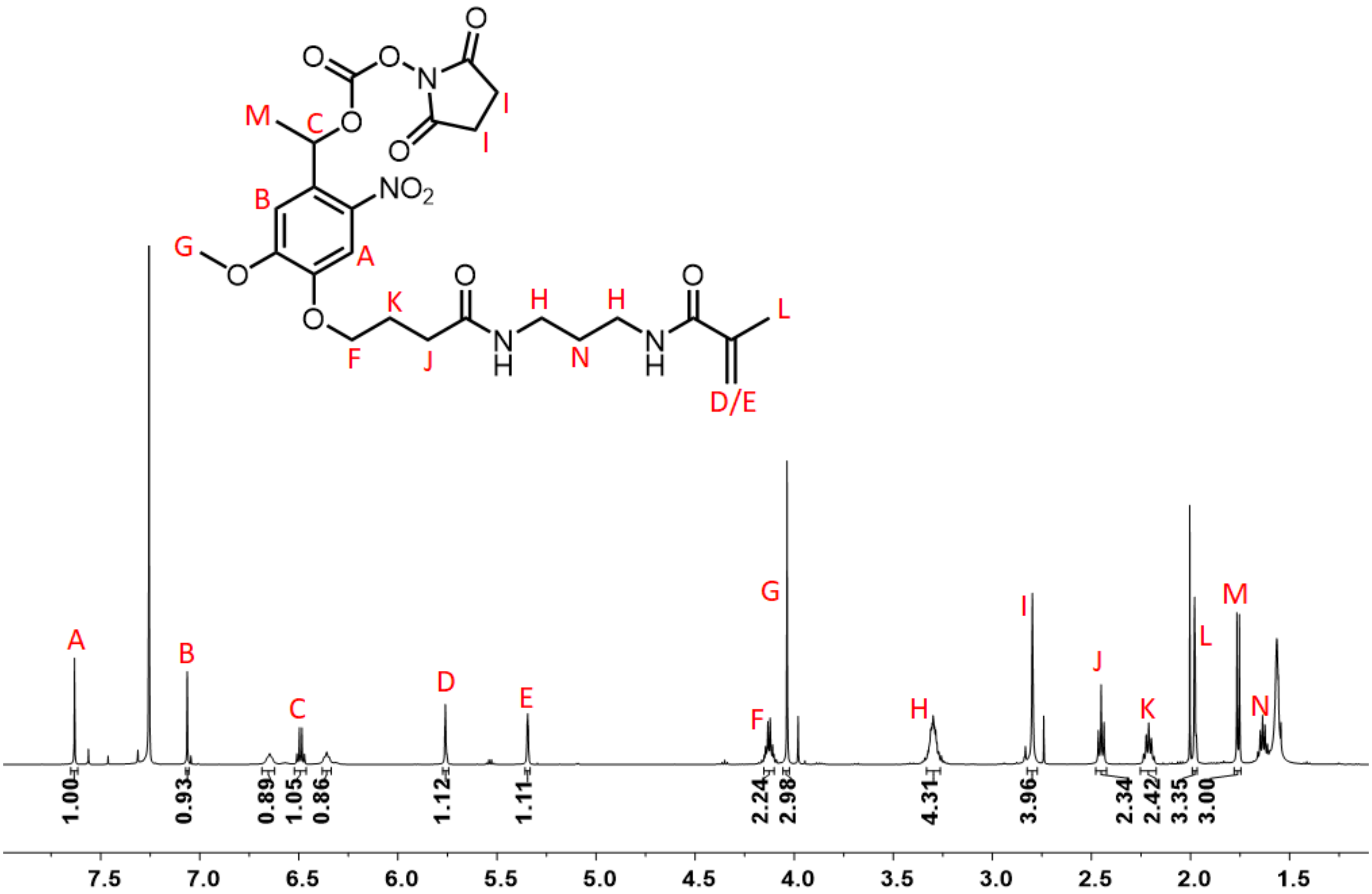

NO2
M
C
B
A
G
K
H
H
L
F
J
N
D/E
I
I
A
B
C
D
E
G
F
H
I
J
K
L
M
N
1.00
0.93
0.89
1.05
0.86
1.12
1.11
2.24
2.98
4.31
3.96
2.34
2.42
3.35
3.00
7.5
7.0
6.5
6.0
5.5
5.0
4.5
4.0
3.5
3.0
2.5
2.0
1.5

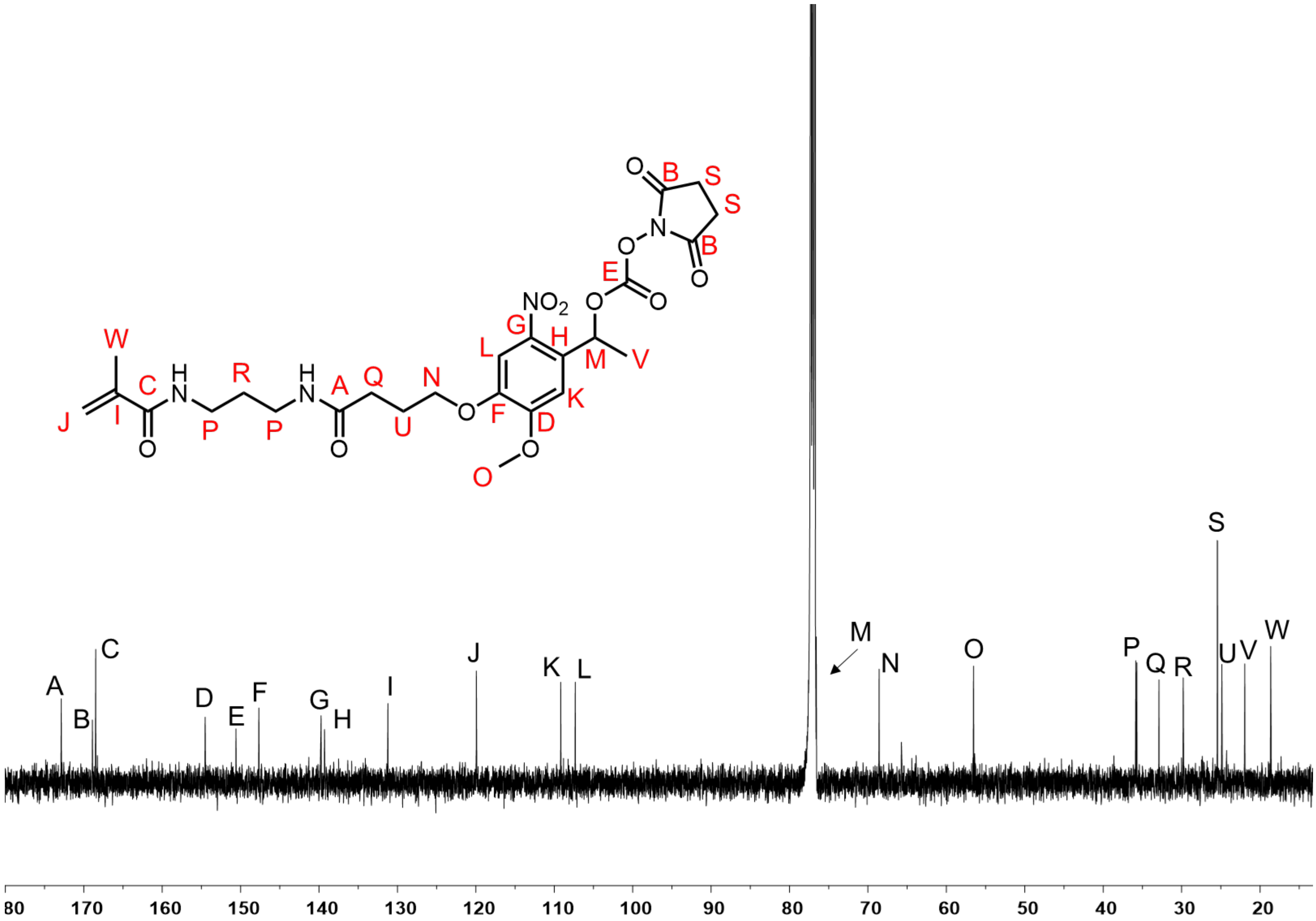
A
B
C
D
E
F
G
H
I
J
K
L
M
N
O
P
Q
R
S
U
V
W
NO2
80
170
160
150
140
130
120
110
100
90
80
70
60
50
40
30
20
